# Natural product scaffolds as inspiration for the design and synthesis of 20S human proteasome inhibitors

**DOI:** 10.1039/d0cb00111b

**Published:** 2020-09-16

**Authors:** Grace E. Hubbell, Jetze J. Tepe

**Affiliations:** Department of Chemistry, Michigan State University East Lansing MI 48823 USA tepe@chemistry.msu.edu

## Abstract

The 20S proteasome is a valuable target for the treatment of a number of diseases including cancer, neurodegenerative disease, and parasitic infection. In an effort to discover novel inhibitors of the 20S proteasome, many reseaarchers have looked to natural products as potential leads for drug discovery. The following review discusses the efforts made in the field to isolate and identify natural products as inhibitors of the proteasome. In addition, we describe some of the modifications made to natural products in order to discover more potent and selective inhibitors for potential disease treatment.

## Abstract 2

The 20S proteasome is a valuable target for the treatment of a number of diseases including cancer, neurodegenerative disease, and parasitic infection.

## Graphical abstract

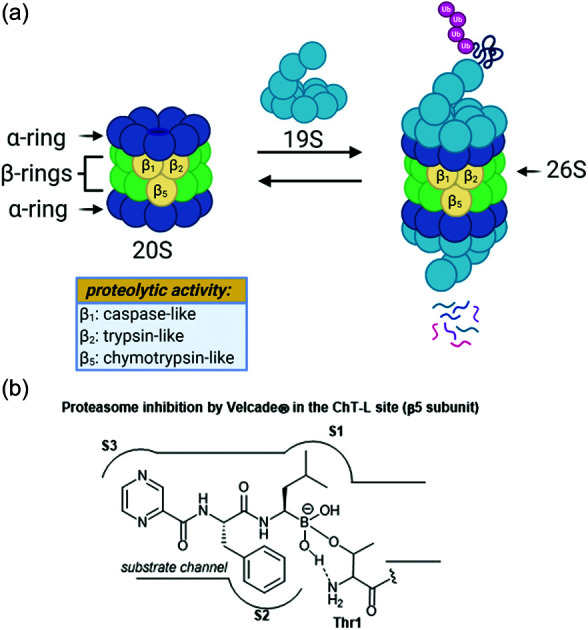

## Introduction

**Fig. 1:**
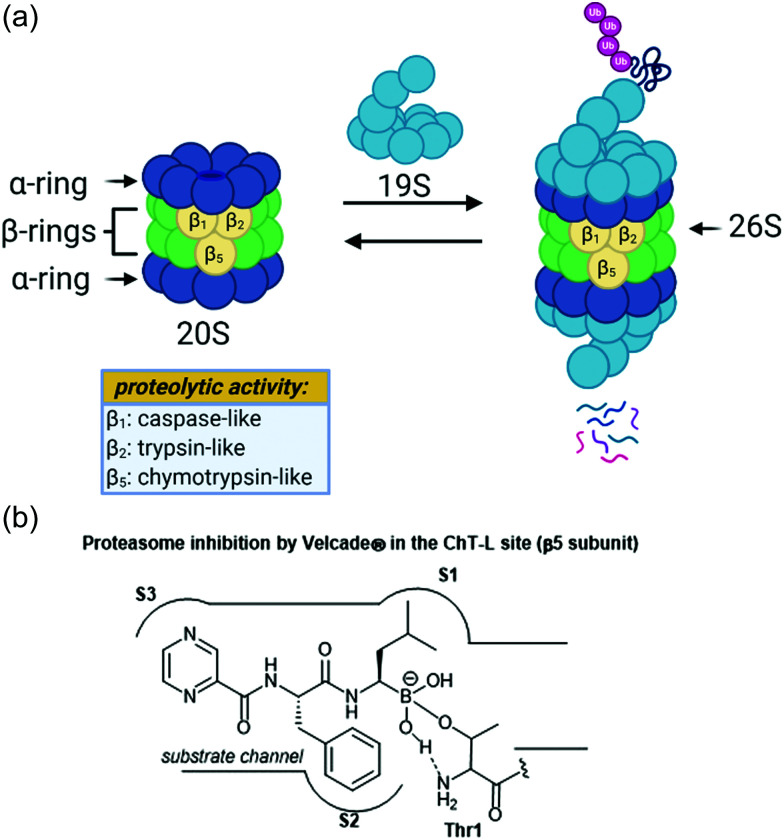
(a) The assembly of the 26S proteasome complex and depiction of proteolysis are shown above. The proteolytic activities of the beta subunits are also shown. (b) The mechanism of action of Velcade® towards the chymotrypsin-like site of the proteasome is shown. The boronic acid undergoes reversible nucleophilic attack by the Thr1 residue within the catalytic site.

The ubiquitin-proteasome pathway plays an integral role in maintaining homeostasis in eukaryotic cells.^1^ The 26S proteasome is a 2.5 MDa threonine protease which is responsible for the degradation of redundant proteins in a cell into oligopeptides for further processing by other pathways (Fig. 1). Ubiquitin-proteasome-mediated degradation has been implicated in the regulation of many signalling proteins including cyclins (involved in cell-cycle progression), p53 (tumor suppressor),^2^ and IκBα (inhibitor of transcription factor NF-κB).^3^

The 26S proteasome is comprised of one or two 19S regulatory particles (RP) which associate with the 700 kDA barrel-like 20S core particle (CP).^4^ The 20S core particle is composed of four stacked heptameric rings in an α_1–7_β_1–7_β_1–7_α_1–7_ arrangement.^5^ The outer α-rings are responsible for recognition of the regulatory particles, and the inner β-rings house the catalytic activity of the protease. The β-rings each consist of seven unique subunits, and three of these subunits (β1, β2, and β5) are responsible for the proteolytic activity of the proteasome. Binding of the 19S caps to the CP facilitates gate-opening, which in turn allows for the proteolytic degradation of polyubiquitinylated proteins. The 19S caps are responsible not only for this gate-opening,^6^ but also the recognition of polyubiquitinylated proteins, unravelling of these into linear peptides, and feeding the peptides into the catalytic chamber.^7^ Once fed into the chamber, unravelled proteins are cleaved into oligopeptides by the catalytic sites, which all proteolytically cleave the peptide chains with the aid of an N-terminal threonine residue (Thr1). Although all three catalytic sites use a nucleophilic threonine to carry out their activity, slight differences between their substrate channels allow for preference towards cleavage of specific peptide residues. The proteasome exhibits caspase-like (C-L, peptidyl-glutamidyl peptide hydrolysing), trypsin-like (T-L) and chymotrypsin-like (ChT-L) activities which are carried out by its β1, β2, and β5 subunits, respectively.^8–10^ The first 20S proteasome structure determined was that of the archaebacterium *Thermoplasma acidophilum* in 1995,^5^ and many eukaryotic proteasomes have since been characterized. Structural similarity between eukaryotic proteasomes is highly conserved, which has allowed for the use of models such as the yeast 20S proteasome^11^ and mammalian 20S proteasome^12,13^ for identification of molecules which exhibit modulatory activity against the proteasome. These models have been especially useful considering that while their X-ray crystal structures were solved throughout the 1990s and early 2000s, which has facilitated inhibitor design greatly, the X-ray crystal structure of the human 20S proteasome was only recently solved in 2015.^14^ Two other core particles have also been identified as the immunoproteasome^15^ and the thymoproteasome.^16^ The differences of selectivity between these are governed by slight differences in the topology within their substrate channels.^17^

Due to its integral role in maintaining cell homeostasis, modulation of the activity of the 20S proteasome has been considered as a potential way to treat several diseases including cancer, autoimmune disease, and neurodegeneration.^18–20^ Additionally, the proteasome of parasitic species has recently been targeted in the treatment of parasitic diseases.^21^ Inhibition of the proteasome has been implicated as a veritable strategy for the treatment of certain cancers, given that inhibition leads to ER stress and apoptosis.^22^ The invention of the proteasome-inhibitor Velcade® (bortezomib) is perhaps the best representation of how targeting the ubiquitin-proteasome pathway can lead to the treatment of some cancers. This dipeptide boronate was approved by the FDA in 2003 as a treatment for multiple myeloma, and its mechanism of action is through direct inhibition of the ChT-L activity of the proteasome.^23^ Activation of the proteasome has also been considered for the treatment of neurodegenerative diseases such as Parkinson's and Alzheimer's disease.^24–26^

Following the discovery of the 26S proteasome complex in the mid-1990s, researchers focused upon the development of novel inhibitors of the 20S proteasome. Most compounds which have been designed as inhibitors of the 20S proteasome are built upon peptide-based scaffolds. Peptide-based scaffolds contain optimized amino acid residues to mimic substrates of the 20S proteasome and improve recognition by the different catalytic subunits. The residues also participate in hydrogen-bonding and hydrophobic interactions to optimize interaction with the substrate channel. Specific design of the peptide chain has allowed for the generation of subsite-specific inhibitors of the proteasome. The design of such inhibitors is based upon the inherent preference of the C-L, T-L and ChT-L activities towards acidic, basic, and hydrophobic residues, respectively. Additionally, these compounds typically contain an electrophilic warhead at their C-terminus which interacts directly with the N-terminal threonine hydroxyl residue of proteolytic sites to block their catalytic activity. The first synthetic proteasome inhibitors were peptide aldehydes^27^ which had been shown to inhibit several types of proteases including serine and cysteine proteases. These peptide aldehydes act as reversible covalent inhibitors of the 20S proteasome, forming a hemiacetal with the nucleophilic threonine residue. Design of more selective inhibitors stemmed from alteration of the electrophilic warhead, leading to the eventual inclusion of boronic acids, vinyl sulfones,^28^ and α-ketoaldehydes^29^ within the scaffolds of synthetic inhibitors of the 20S proteasome. The interaction between covalent inhibitors and the catalytic sites is demonstrated in Fig. 1b with bortezomib. The substrate channel accommodates the peptide side chains of the molecule much like it would a peptide substrate. The electrophilic boronic acid moiety at the terminus of the molecule is susceptible to nucleophilic attack by the Thr1 residue of the catalytic site. This electrophile undergoes reversible nucleophilic addition by the amino acid, and the resulting charged species is stabilized through hydrophobic interactions and hydrogen-bonding interactions.^30^ Noncovalent inhibitors typically capitalize upon purely hydrophobic and hydrogen-bonding interactions to confer inhibition.

Nature is an attractive source for the identification of novel proteasome inhibitors: the inherent stereochemical complexity of many natural products may allow researchers insight into unique interactions relative to the established clinically available inhibitors. A variety of methods exist for mining nature for identification of novel proteasome inhibitors including the use of fluorogenic peptide assay, site-specific probes, pathway-specific accumulation assay and NMR spectroscopic assay.^31^ These methods have been especially helpful in regards to identification of natural product inhibitors, as some may be utilized with crude mixtures before isolation. Examples of the use of these methods are included in the review. Elucidation of the mechanisms of action by natural products through the methods of X-ray crystallography and computational docking allow for the design of more potent and selective inhibitors based upon the natural product scaffold. The focus of this review is to discuss several classes of natural products which have been identified for their inhibitory activity towards the 20S proteasome, their mechanism of action, and the efforts taken to optimize these scaffolds for potency and selectivity towards human 20S proteasome inhibition.

## Natural product scaffolds that inhibit the 20S proteasome

### Peptide-aldehydes

**Chart 1:**
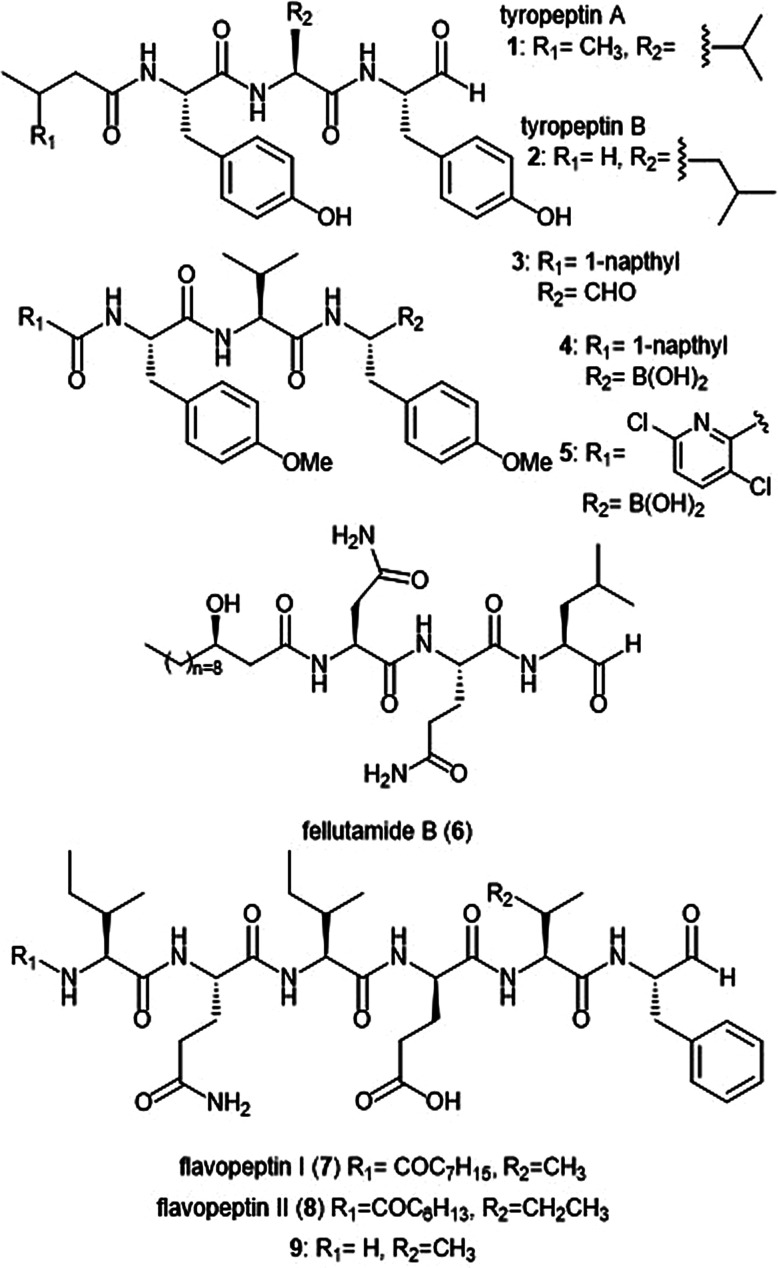
Peptide aldehyde natural products and derivatives that inhibit the 20S proteasome are shown.

Several families of peptide aldehyde natural products have been identified for their inhibitory activity towards the 20S proteasome (Chart 1). The first established natural product-based peptide aldehyde inhibitors tyropeptins A and B are products of the actinomycete microbe *Kitasatospora* sp. MK993-dF2, a strain originally discovered in a soil sample from Kami-gun, Japan.^32,33^ After extensive spectroscopic analysis, the structures of the tyropeptins A and B were determined as isovaleryl-l-tyrosyl-l-valyl-dl-tyrosinal and *n*-butyryl-l-tyrosyl-l-leucyl-dl-tyrosinal, respectively. For confirmation of their structures, the total syntheses of the natural products were completed and compared with the isolated samples. The peptide backbone and reactive aldehyde moiety had already been established as a promising proteasome inhibiting scaffold,^34^ and therefore the potential biological activities of tyropeptins A and B were of interest to researchers. Tyropeptin A (**1**) competitively inhibits the ChT-L and T-L activities of the 20S proteasome with IC_50_ values of 0.20 μM and 2.9 μM, respectively. Tyropeptin B (**2**) is a weaker competitive inhibitor of the 20S proteasome, with IC_50_ values for the ChT-L and T-L sites of 0.39 μM and 7.8 μM, respectively. Tyropeptin A also exhibited cytotoxicity against HeLa S3 human cervical cells and HL-60 promyelocytic leukemia cells with IC_50_ values of 33.2 μM and 8.6 μM, respectively. Derivatives of tyropeptin A were designed by Momose *et al.* using computational methods.^35,36^ At the time, the crystal structure of the human 20S proteasome had not yet been solved; however, the crystal structure of the bovine 20S proteasome had been determined and was therefore used as a model.^12,13^ Tyropeptin A was believed to undergo nucleophilic attack by the Thr1O^γ^ residue of the active site to form a hemiacetal, and its side chains interact with the subpockets to form an antiparallel β-sheet. Introduction of bulky aromatic groups at the N-terminus enhanced inhibitory activity towards the ChT-L site of the proteasome. Optimization led to TP-110 (**3**), an N-terminal naphthyl derivative with methylated hydroxy positions (IC_50_: 0.027 μM). TP-110 exhibited improved growth inhibitory activity against cancer cells, likely due to increased hydrophobicity of the molecule to improve cell permeability. In subsequent *in vitro* studies, TP-110 also repressed the chymotrypsin-like activity of the proteasome in human prostate cancer PC-3 cells, inhibiting the proteasome activity by 56% with 0.16 μM.^37^ Furthermore, TP-110 induces apoptosis in the prostate cancer cell line. Substitution of the formyl group in TP-110 with boronates resulted in enhanced inhibitory potency towards the ChT-L site.^38^ Optimization of this scaffold led to boronate **4**, which bore an N-terminal 3,6-dichloro-2-pyridyl substituent and exhibited comparable inhibitory potency towards the chymotrypsin-like activity of the proteasome (IC_50_: 0.053 μM) to that of bortezomib.^39^ Analog **4** also displayed good cytotoxic activity towards the RPMI8226 multiple myeloma cells, and was chosen for further *in vitro* and solid tumor studies.^40^

Fellutamide B (**5**) was originally isolated from *Penicillium fellutanium*, a fungus which is found in the gastrointestinal tract of the marine fish *Apogon endekataenia*.^41^ Fellutamide B demonstrated potent cytotoxicity against murine leukemia P388 (IC_50_: 0.18 μM) and L1210 (IC_50_: 1.26 μM) cells, as well as human epidermoid carcinoma KB cells (IC_50_: 1.26 μM) *in vitro*, though the mechanism of cytotoxicity was not established. The total synthesis of fellutamide B was first achieved by the Crews group.^42^ Since then, other groups have also set out to achieve total syntheses of not only fellutamide B, but other members of the fellutamide family as well.^43–45^ Fellutamide B potently inhibits the chymotrypsin-like site (IC_50_: 9.4 ± 2.5 nM), but the trypsin-like and caspase-like sites to a lesser extent (IC_50_: 2.0 ± 0.4 μM and 1.2 ± 0.8 μM, respectively).^46^*In vitro* studies revealed the ability of the natural product to inhibit the proteasome within cells, as treatment of L–M mouse fibroblasts resulted in accumulation of ubiquitinated proteins.

The flavopeptin class of peptide aldehydes was discovered with the help of proteomics.^47^ The Kelleher group utilized the method of Proteomic Investigation of Secondary Metabolism (PrISM) to screen *Streptomyces* species for non-ribosomal peptide synthases (NRPS) responsible for secondary metabolite synthesis. Using the PrISM approach, the group was able to identify a novel NRPS gene cluster from *Streptomyces* sp. NRRL F-6652. With the help of bioinformatics analysis and metabolomics analysis, six flavopeptins were discovered as the products of this gene cluster. Flavopeptins I (**6**) and II (**7**) were synthesized using solid phase peptide synthesis (SPPS), along with the N-terminal amine derivative **8**. Due to the similarity of the flavopeptins to other established inhibitors, the natural products were tested for their inhibitory activity towards the ChT-L and C-L sites of the 20S proteasome. Flavopeptins I and II are low micromolar inhibitors of both sites, and the terminal amine derivative **8** exhibits submicromolar IC_50_ values for both sites. The flavopeptins were also tested for their cytotoxicity against the multiple myeloma cell lines MM.M1S and FR4, as well as the histiocytic lymphoma cell line, U-937. Flavopeptin I displayed values of IC_50_: ∼35 and 13 μM for the MM cell lines and the lymphoma line, respectively. The N-terminal amine derivative **8** displayed no cytotoxic activity. The lack of activity is believed to be due to lower cell permeability and stability as compared to flavopeptins I and II.

### Syrbactins

**Chart 2:**
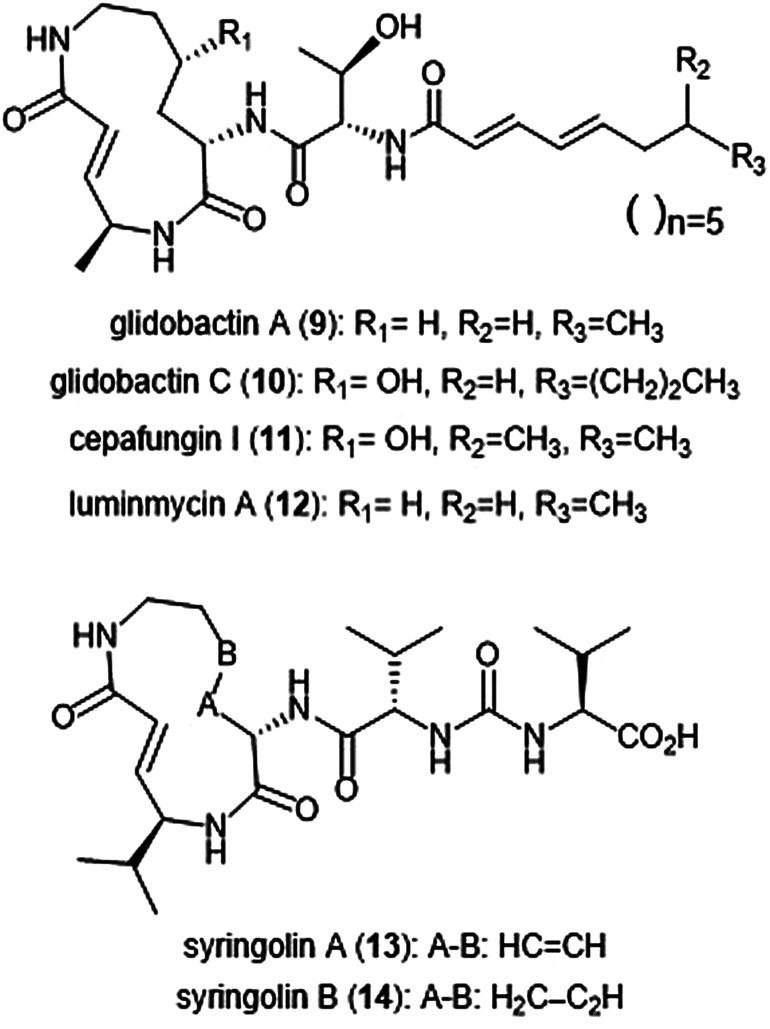
Syrbactin natural products which inhibit the 20S proteasome.

Several syrbactin natural products have also been identified for their proteasome inhibition (Chart 2). Syrbactins are divided into 3 sub-families: syringolins, glidobactins, and cepafungins. These compounds are products of plant pathogens, and their similarities include a macrocyclic lactam with an electrophilic α,β-unsaturated carboxamide. Glidobactins (A–H) are natural product syrbactins that are produced by the bacterial strain *Polyangium brachysporum* sp. nov. K481–B101.^48–51^ Glidobactins A–C are cytotoxic against melanotic melanoma B16 cells as well as human colon cancer HCT-116 cells, and exhibit potent *in vivo* antitumoral activity towards P388 leukemia in mice.^48^ Further modification of the glidobactin A structure by Oka *et al.* led to the preparation of several derivatives, which were also evaluated for their antitumoral activity.^51^ Glidobactin A (**9**) was later identified as a nanomolar inhibitor of the ChT-L site of the 20S proteasome, with a *K*_i_ value of 49 ± 5.4 nM.^52^ The compound also inhibited T-L activity of the 20S proteasome, albeit at considerably higher concentrations (2000 ± 600 nM, *n* = 6). The mechanism of inhibition by syrbactins was determined through elucidation of the X-ray crystal structure of the 20S proteasome in complex with glidobactin A and syringolin A. The α,β-unsaturated moiety of glidobactin A undergoes a nucleophilic Michael-type 1,4-addition by the Thr1O^γ^ of the chymotrypsin-like and trypsin-like sites of the proteasome. The lipophilic alkyl chain of glidobactin A contributes to its inhibitory activity and served as inspiration in the development of novel syrbactin proteasome inhibitors; the activity of these analogs is discussed in a later section.

Glidobactin C (**10**) was recently identified as an inhibitor of the human constitutive and immunoproteasomes through a competitive metabolite profiling technique by the Böttcher group.^53^ Glidobactin C exhibited *in vitro* inhibitory activity towards the ChT-L and T-L sites of the human constitutive proteasome (IC_50_: 2.9 ± 2.2 nM and 2.4 ± 2.8 nM, respectively). The compound also inhibits the human immunoproteasome (ChT-L IC_50_: 7.1 ± 5.3 nM; T-L IC_50_: 2.5 ± 2.0 nM). The two additional methylene groups of **10** relative to **9** are thought to improve its ability to penetrate the cell membrane, resulting in greater activity in cell assays.

The cepafungins were observed as products of the bacteria *Pseudomonas* sp. CB-3 by Shoji *et al.*^54^ Cepafungins I, II, and III were identified as acylpeptide antibiotics which all contain a distinct 12-membered macrolactam ring.^55^ The macrolactam ring of all three compounds is comprised of the two amino acids γ-hydroxylysine and 4-amino-2-pentenoic acid, and the distinguishing feature among each is a variable fatty acyl tail at the N-terminus of the peptide. Based upon their analysis, the group concluded that the structure of cepafungin II is identical to that of known natural product glidobactin A. The complex of all three compounds displayed a moderate ability to prolong the survival period of mice which bear murine lymphatic leukemia P388 cells. Cepafungin I (**11**) was identified as a proteasome inhibitor in later studies: Stein *et al.* developed an NMR-based proteasome assay to identify natural product inhibitors of the yeast 20S proteasome.^56^ Cepafungin I potently inhibits the ChT-L activity of the proteasome (IC_50_: 4 nM), five times more potent than the known inhibitor glidobactin A (IC_50_: 19 nM). Cepafungin I also inhibits the T-L activity of the proteasome with an IC_50_ value of 24 nM. The improved inhibitory activity of cepafungin I as compared to glidobactin A is believed to be due to the increased stabilization of the fatty acyl tail through van der Waals interactions.

Luminmycin A (**12**) is another natural product of the syrbactin family which has recently gained attention as a potential proteasome inhibitor. A deoxy derivative of glidobactin A, luminmycin A contains the hallmark 12-membered macrolactam ring responsible for the inhibitory activity of the syrbactin family towards the human 20S proteasome. Luminmycin A was identified as a metabolite of a silenced gene cluster *plu1881–plu1887* of *Photorhabdus luminescens*, and the associated gene cluster was utilized to produce luminmycin A using the method of heterologous expression.^57^ The natural product was also observed as a metabolite of crickets following their infection with *Photorhabdus asymbiotica*.^58^ Luminmycin A and other natural products belonging to the luminmycin class were successfully isolated following heterologous expression of the same gene cluster, using *E. coli* as host.^59,60^ Recently, luminmycin A was successfully synthesized in the laboratory by Servatius *et al.*^61^ Luminmycin A inhibits the constitutive proteasome (CP) as well as the immunoproteasome (IP), (ChT-L IC_50_: 0.039 ± 0.002 μM (CP), 0.016 ± 0.006 μM (IP); T-L 0.026 ± 0.008 μM (CP), 0.017 ± 0.0016 μM (IP)).^53^ The natural product also exhibits cytotoxic activity against human carcinoma HCT-116 cells (IC_50_: 91.8 nM).

The syringolin family of natural products were identified as secondary metabolites of the pathogen *Pseudomonas syringae* pv. *syringae*, a non-host pathogen of the *Oryza sativa* rice plants.^62,63^ Syringolin A was identified as an inhibitor of the human proteasome in 2008, thus prompting interest in the successful completion of its total synthesis by several research groups. The total syntheses of syringolins A and B were first completed in 2009, with many more syntheses to follow.^64–66^*In vitro* enzymatic assays revealed the inhibitory activities of syringolins A and B toward the 20S proteasome. Syringolin A (**13**) inhibits the ChT-L and C-L activities of the 20S proteasome (*K*_i_: 1.1 ± 0.179 μM, and 10.3 ± 1.4 μM, respectively). The macrolactam binds covalently to the 20S proteasome by direct interaction with Thr1O^γ^ within the active sites.^52,67^ The α,β-unsaturated carboxamide of syringolin A is rendered especially electrophilic due to the ring strain of the macrocycle resulting from the presence of two *trans* alkenes; upon 1,4-Michael addition of Thr1O^γ^, this ring strain is relieved. In comparison, syringolin B (**14**), which lacks one of the *trans* alkenes, is a significantly less potent inhibitor of the 20S proteasome (*K*_i_: 7.7 ± 2.3 μM (ChT-L); 107.8 ± 39.2 μM (T-L).

**Chart 3:**
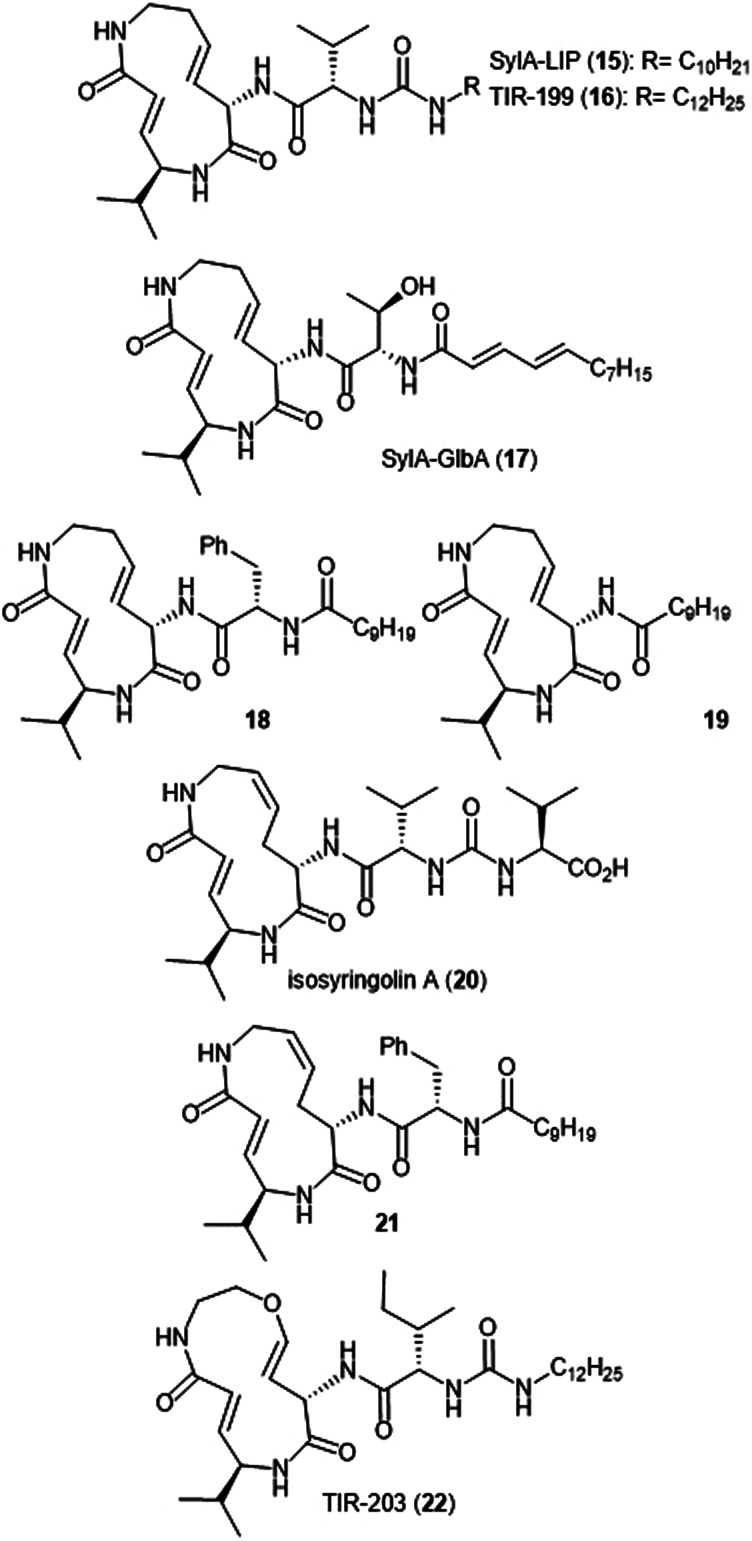
Proteasome inhibitors based on the syringolin-A scaffold are shown.

Additional derivatization of the syringolin A scaffold has been pursued by several groups (Chart 3). Alteration of the N-terminus of syringolin A from a carboxylic acid to a lipophilic tail led to the production of SylA-LIP **15**, which exhibits inhibitory activity towards all three catalytic sites of the proteasome (*K*_i_: 8.65 ± 1.33 nM (ChT-L); 79.6 ± 29.3 nM (T-L); and 943 ± 100 nM (C-L)).^68^ TIR-199 (**16**) contains a slightly longer lipophilic tail than SylA-LIP and inhibits the ChT-L (*K*_i_: 18 nM) and T-L (*K*_i_: 194 nM) sites of the human proteasome in *in vitro* enzymatic assays.^69^ TIR-199 also inhibits the proteasome in multiple myeloma cell line MM1.RL and neuroblastoma cell line MYCN2 and has demonstrated exciting cytotoxic activity against bortezomib-resistant cell lines (MM1.S BzR and U266 BzR), indicating it can overcome bortezomib resistance *in vivo.*^70^ Clerc *et al.* further investigated the effects of substitution at the N-terminus through the synthesis of a syringolin A–glidobactin A hybrid **17**.^71^ The inhibitory activity and subsite selectivity of the analogue was tested using competitive activity-based protein profiling (ABPP) in HEK cell lysates and living cells, compared to known syrbactin inhibitors. Further *in vitro* studies revealed that **17** inhibits all three catalytic sites of the 20S proteasome (*K*_i_: 12.5 ± 1.5 nM (ChT-L), 136.9 ± 12.4 nM (T-L), 3.7 ± 1.2 μM (Casp-L)).^72^ Cell culture-based experiments from the same study revealed that the analogue carries out inhibition of the proteasome in several cancer cell lines. Introduction of hydrophobic sidechains allowed the group to target the S3 subpocket of the β5 subunit for inhibition. Compound **18**, which contained a benzyl substituent and lipophilic side chain, displayed strong inhibitory activity against the ChT-L site of the proteasome (*K*_i_′: 0.12 nM) and cytotoxicity towards human RPMI8226 multiple myeloma cells (IC_50_: 2.2 nM).^66^ Further optimization of **18** has focused on improving cytotoxicity.^73^

Simplification of the syringolin A scaffold has also been a focus of research by the Ichikawa group. One report focused on analogues that exhibited proteasome inhibition and cytotoxicity against cancer cells by targeting a different intermolecular H-bonding interaction.^74^ The N-terminal amide of analogue **18** is believed to participate in a hydrogen-bonding interaction with Asp114 (β6), similarly to its parent syringolin A. The group hypothesized that removal of the N-terminal amide group and replacement with a longer alkyl chain may allow for a switching in the hydrogen-bonding interactions within the β5 subunit and allow for a novel interaction between the substrate and Ala49. The hypothesis was verified through the synthesis of analogue **19**, which displayed nanomolar inhibitory activity towards the ChT-L site of the proteasome (IC_50_: 107 nM). Additionally, analogue **19** exhibits nanomolar growth inhibitory activity against several cancer cell lines.

Alteration of the macrocycle structure was also explored. Researchers hypothesized that a more accessible alkene isomer with a similar three-dimensional structure might be also be able to carry out inhibitory activity. The group synthesized isosyringolin A (**20**) and its analogue **21**, which both exhibited inhibition towards the ChT-L site (*K*_i_ = 590 nM; 1.53 nM, respectively).^75^ Compound **21** also exhibited potent cytotoxicity against OPM-2 human myeloma cancer cells (IC_50_: 6.7 nM) and bortezomib-resistant OPM-2 cells (IC_50_: 60 nM), displaying greater cytotoxicity as compared to bortezomib (IC_50_: 146 nM). Expansion of the syringolin A ring by Ibarra-Rivera *et al.* led to oxa-SylA-LIP (**22**), which displays potent cytotoxicity against the MYCN-2 cells (IC_50_: 0.4 μM) and also inhibits the proteasome in cell-based studies.^76,77^

**Chart 4:**
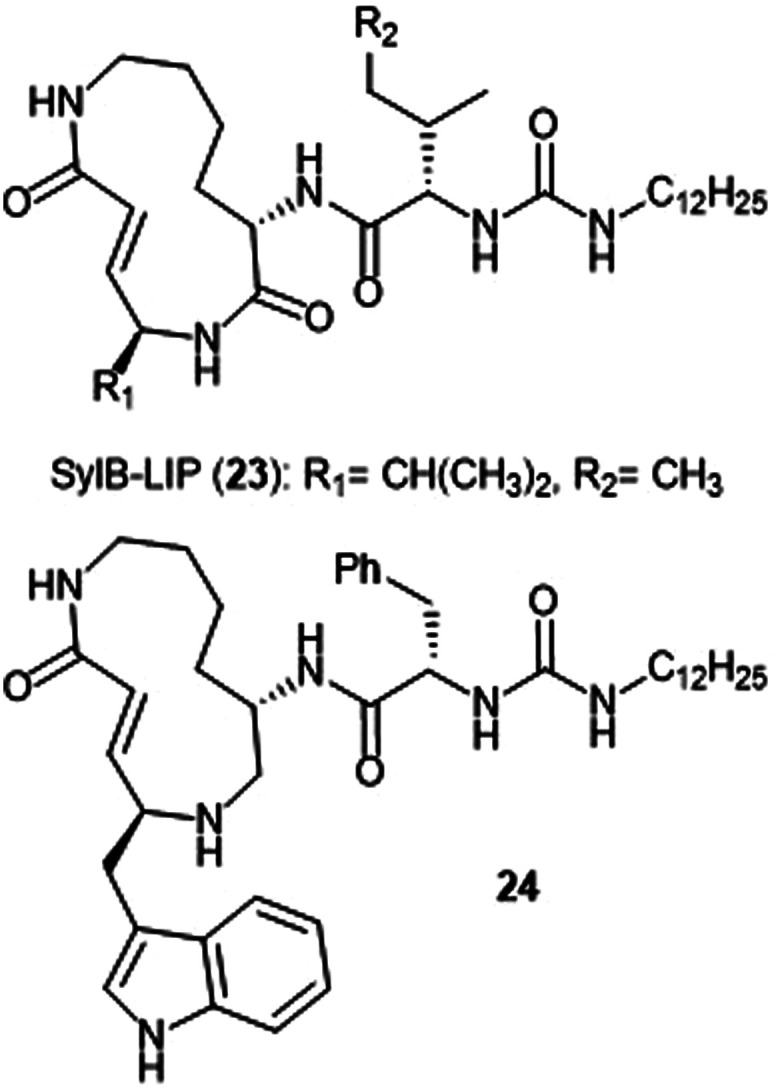
Syringolin-B based inhibitors of the 20S proteasome are depicted.

Analogue development based upon the syringolin B scaffold has also been investigated by several research groups (Chart 4). For example, the SylB-LIP analogue (**23**) was developed as a direct comparison to SylA-LIP (**15**).^76^ Totaro *et al.* designed more potent syringolin B analogues with greater inhibition towards the β5 subunit based upon the synthesis of syringolin B by Pirrung *et al.*^65,78^ Introduction of aromatic groups at the P1 and P3 residues in the scaffold led to the discovery of potent inhibitor **24** (second order rate-constant (*k*_in_/*K*_i_): 4305 M^−1^ s^−1^). The analogue also exhibits potent growth inhibition against various leukemia cell lines.

Thiasyrbactin analogues with a sulfur-for-carbon substitution within the macrolactam ring were synthesized with varying levels of sulfur oxidation. These analogues were reported for their activity towards both the constitutive and immunoproteasomes, more selectively inhibiting the T-L site of the immunoproteasome.^79^ The biological activity of the analogues was further examined through cytotoxicity experiments with numerous neuroblastoma cell lines. The thiasyrbactin scaffold has established itself as a drug-like starting point for inhibition of the immunoproteasome.

### Macrocyclic peptides

**Chart 5:**
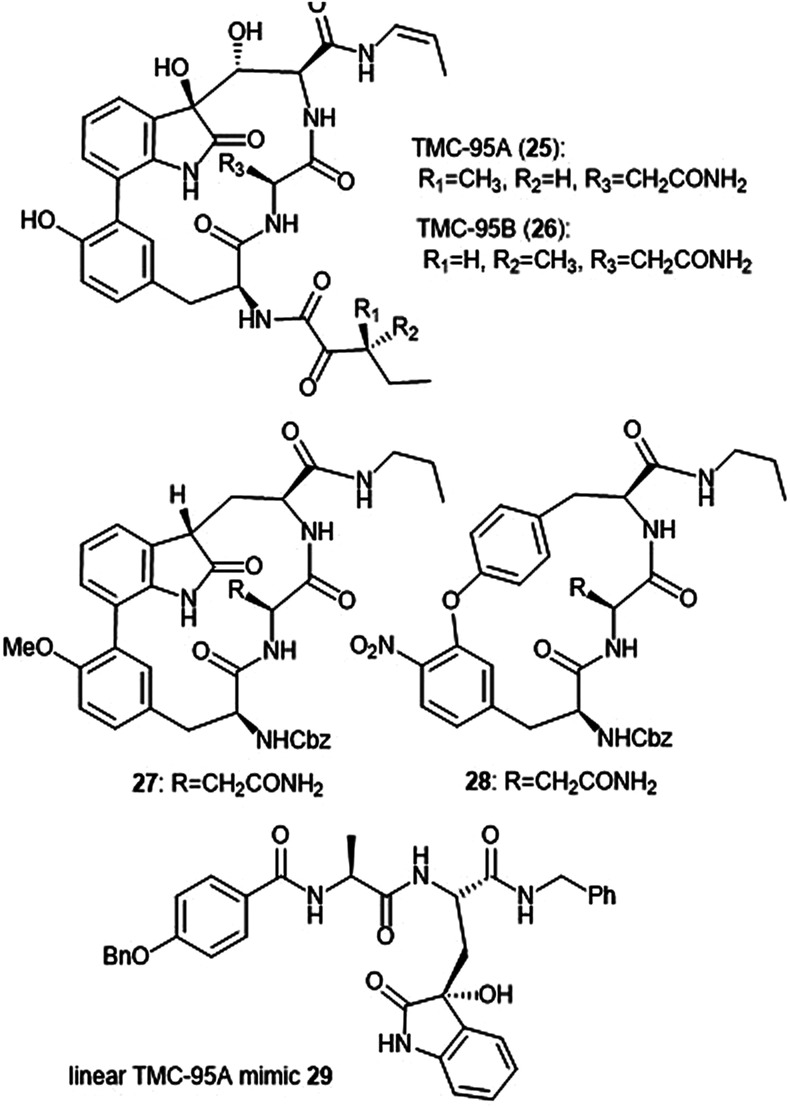
The TMC-95 natural products and subsequent inhibitors developed based upon the scaffold are shown.

Several classes of macrocyclic peptides have been identified for their inhibitory activity towards the proteasome. Perhaps the most explored class has been the TMC cyclic peptides (Chart 5). TMC-95 A, B, C, and D were isolated from the fermentation broth of *Apiospora montagnei* Sacc. TC 1093 by Koguchi *et al.* while the group was screening for 20S proteasome inhibitors.^80,81^ Among all of the TMC cyclic peptides, TMC-95 A (**25**) and B (**26**) were identified as specific inhibitors of the 20S proteasome, displaying activity against both the ChT-L (IC_50_: 5.4 nM (TMC-95 A) and 8.7 nM (TMC-95 B)) and T-L (IC_50_: 200 nM (TMC-95 A) and 490 nM (TMC-95 B)) subunits. TMC-95A further demonstrated cytotoxic activities against HCT-116 and HL-60 cells with IC_50_ values of 4.4 μM and 9.8 μM, respectively. The X-ray crystal structure of the yCP:TMC-95A complex was later solved by Groll *et al.* (2.9 Å resolution), revealing that the macrocycle reversibly inhibits the 20S CP through non-covalent interactions within the catalytic core.^82^ The specificity of the natural product for the chymotrypsin-like site is believed to be due to greater interaction with the S3 specificity pocket.

Due to the biological activity and overall complexity of the TMC-95 natural products, many groups sought to undertake their synthesis.^83–86^ The first total syntheses of TMC-95A and B were achieved by Lin and Danishefsky in 2002.^83^ The presence of sensitive functional groups in addition to the difficulty in accessing the macrocycle has encouraged subsequent analogue development to be focused on the synthesis of simplified molecules. Kaiser *et al.* synthesized compound **27**, a simplified analogue based upon the minimal requirements for binding as determined from the X-ray crystal structure of TMC-95A/proteasome complex.^87^ The analogue exhibited inhibitory activity against the ChT-L site of the 20S human proteasome (IC_50_: 1.9 μM) as compared to TMC-95A (IC_50_: 0.012 μM).^88^ The same researchers evaluated the effect of additional P′ residues at the C-terminus of TMC-95A in a separate study.^89^ The Danishefsky group established the importance of the enamide moiety at the C-8 position of the molecule;^90^ analogues lacking this displayed at least a 1000-fold less potent inhibition towards the ChT-L site as compared to their natural product parent molecule. Furthermore, alteration of the enamide sidechain also affected inhibitory activity; the presence of a propyl substituent in exchange for a propylene or allyl amide sidechain results in diminished activity. In previous studies, the biaryl moiety of TMC-95A was deemed responsible for the induction and stabilization of a β-type peptide backbone conformation while in complex with the 20S proteasome.^82^ Kaiser *et al.* implemented the substitution of the biaryl heterocycle using a more accessible biaryl ether moiety with the goal of retaining the similar β-type backbone conformation.^91^ The biaryl ether analogue **28** retains inhibitory potency towards the ChT-L site of the yCP (IC_50_: 5.5 μM). Later studies revealed that due to the lack of the oxindole, biaryl ether analogues do not adopt the desired stabilized β-type structure while in complex with the 20S proteasome.^92^ Wilson *et al.* later synthesized macrocyclic peptide aldehydes upon the previously established biaryl ether analogues which are nanomolar inhibitors of the ChT-L site.^93^ Further efforts to access simplified analogues of TMC-95A have eliminated synthesis of the challenging macrocycle altogether through the development of linear peptide mimics of the natural product. The Vidal group—in collaboration with many other research groups—has used this linear TMC-95A approach to synthesize effective inhibitors.^94–99^ In particular, the linear 3-hydroxyoxindole-containing derivatives exhibited subunit selectivity towards the C-L site).^96^ Optimization of the scaffold led to the discovery of potent inhibitor **29**, which inhibited the ChT-L of the constitutive and immunoproteasomes (IC_50_: 7.1 ± 0.2 and 10.2 ± 0.1 nM, respectively).^99^ Dimerized linear TMC-95A mimics using the active derivative **29** scaffold have also been synthesized to evaluate their ability to inhibit multiple active sites at once. Using either PEG spacers^100^ or oligomers of aminohexanoic and adipic acid as spacers,^101^ researchers were able to achieve nanomolar inhibition of the ChT-L sites of the 20S proteasome.

**Chart 6:**
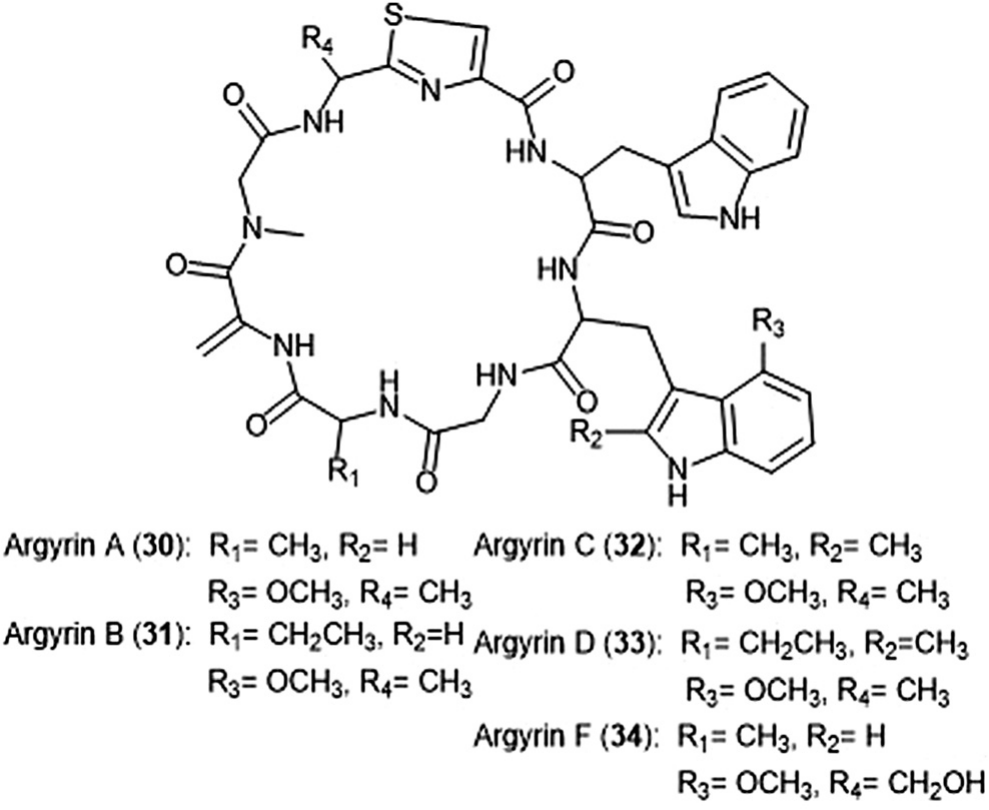
The cyclic argyrin octapeptides which have been identified as inhibtors of the 20S proteasome are depicted.

Argyrins A–H were isolated from the culture broth of myxobacterium *Archangium gephyra* by the Hofle group in 2002.^102,103^ This group of cyclic octapeptides displayed growth inhibitory activity against a variety of mammalian cell lines,^102^ and became a focus of total synthesis for many groups.^104–106^ Argyrins A, B, C, D and F (**30–34**) (Chart 6) were later identified as low nanomolar inhibitors of the human proteasome.^107,108^ Bulow *et al.* established that the methoxy group in Trp2 as well as the *exo*-cyclic methylene group are necessary for the ability to inhibit the proteasome.^108^ The binding mode of the argyrins was later elucidated through NMR spectroscopy and molecular modeling.^109^ Computational inhibition studies using a humanized proteasome model indicated which interactions contributed to argyrin subunit specificity.^110^ Using molecular docking, researchers designed novel argyrin A analogues *in silico* which display increased specificity towards the caspase-like site of the 20S humanized proteasome model. The Loizidou group recently reported the specificity of argyrin B towards the immunoproteasome.^111^

**Chart 7:**
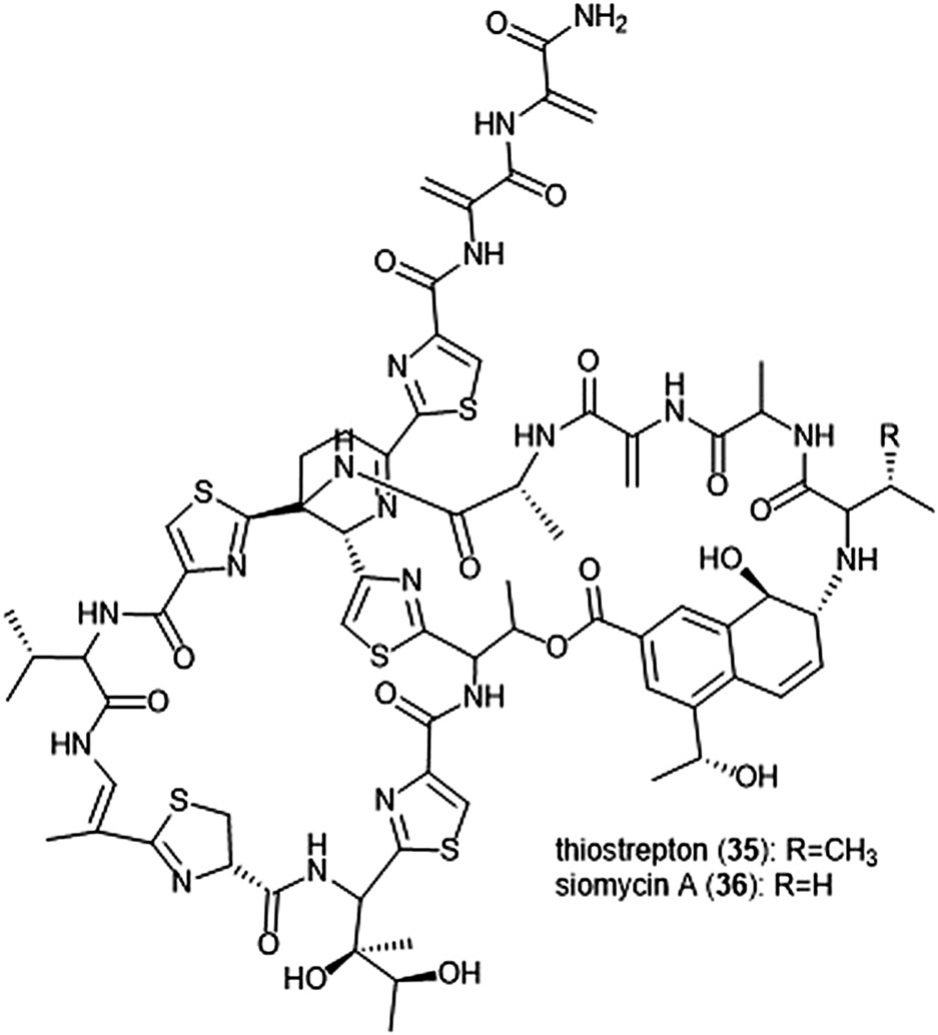
The thiopeptide natural products thiostrepton and siomycin A are shown. The “B-ring” within both contributes to their ability to inhibit the 20S proteasome.

Macrocyclic thiopeptides have also been investigated as inhibitors of the human proteasome. Bhat *et al.* reported that thiopeptides thiostrepton (**35**) and siomycin A (**36**) exhibit inhibitory activity against the 20S proteasome, which in turn contributes to the effects observed on the oncogenic transcription factor Fox1 (Chart 7).^112,113^ The additional “B-ring” present in thiostrepton and siomycin A contributes to their inhibitory activity towards the 20S proteasome.^114^ One avenue of analogue development has focused on targeting the parasite *P. falciparum*, and again illustrated that maintenance of the “B-ring” confers inhibition.^115^ Zhang *et al.* employed the use of an *S. laurentii tsrA* mutant organism to produce Ala2 thiostrepton analogs and additionally evaluated them for their inhibitory activity towards the 20S proteasome.^116^

**Chart 8:**
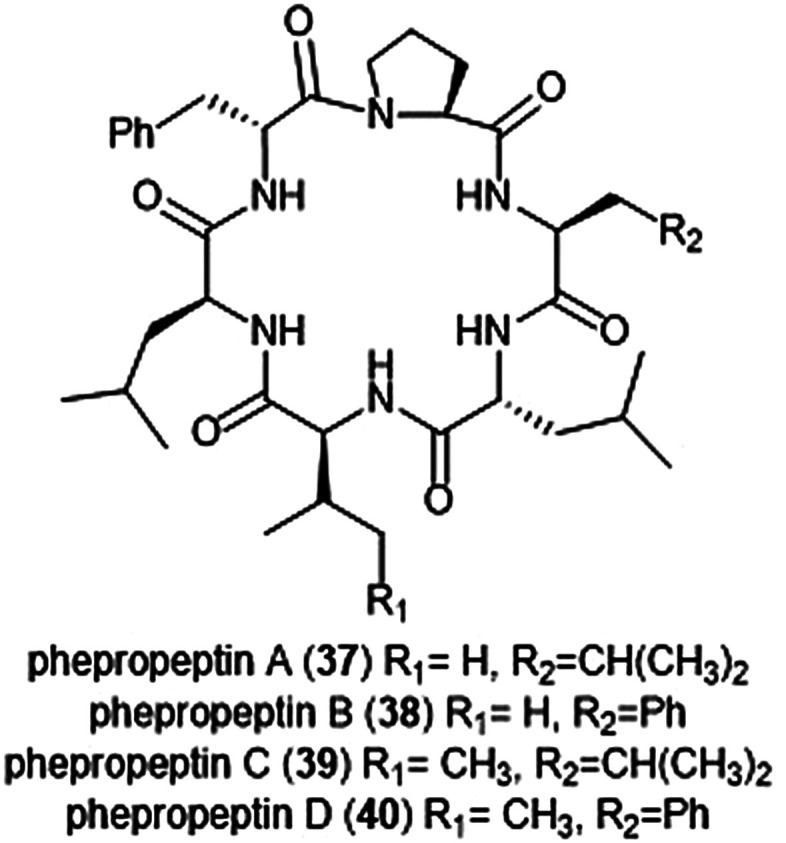
Phepropeptins A–D are depicted; these cyclic peptides are believed to inhibit the proteasome through purely noncovalent interactions.

**Chart 9:**
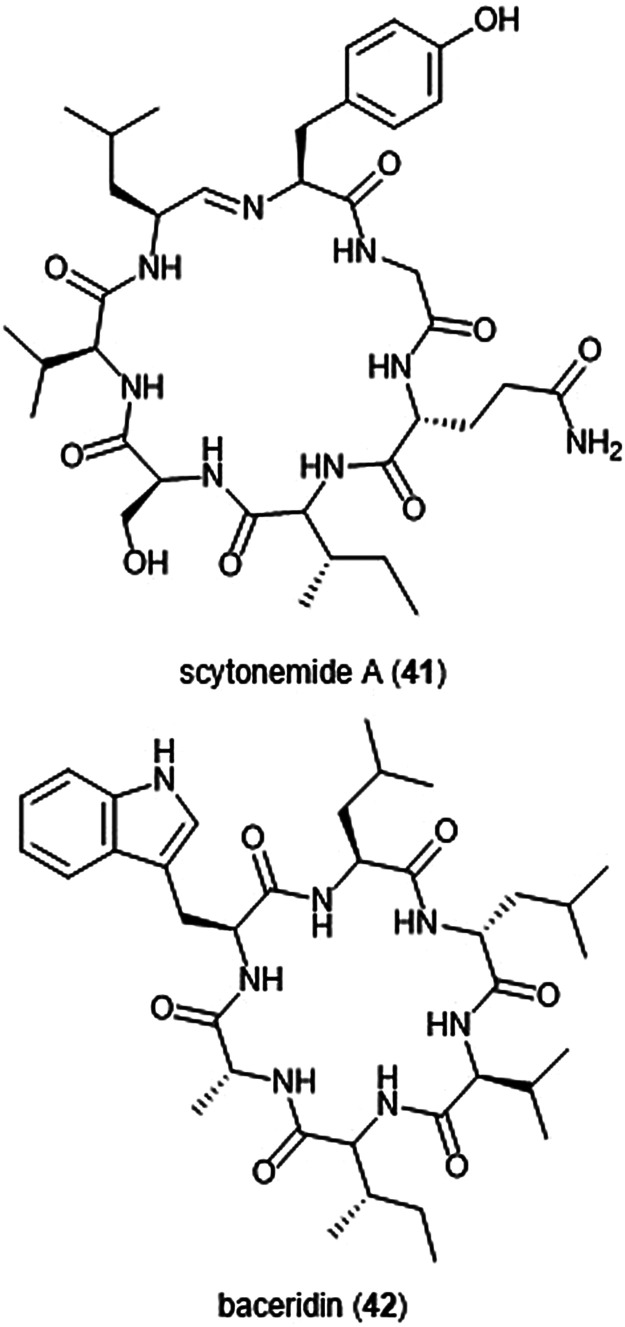
Scytonemide A (**41**) and baceridin (**42**) are cyclic peptides which also inhibit the 20S proteasome.

Several macrocyclic peptide scaffolds that have been identified as inhibitors of the proteasome have yet to be further evaluated through SAR studies (Charts 8 and 9). Among these natural products are the phepropeptins. Phepropeptins A–D (**37–40**) are cyclic hexapeptides isolated by Sekizawa *et al.* from *Streptomyces* sp. MK600-cF7.^117^ All four natural products exhibit inhibitory activity against the β5 subunit (IC_50_: 30.8, 15.3, 17.9, and 10.7 μM, respectively). Because of their stability as cyclic peptides, these natural products were believed to interact with the proteasome through van der Waals and hydrophobic interactions rather than through covalent bond formation. Scytonemide A (**41**)was isolated from the cultured freshwater cyanobacterium *Scytonema hofmannii* (UTEX 1834).^118^ Scytonemide A potently inhibits the ChT-L site of the 20S proteasome (IC_50_: 96 nM). The presence of an imine moiety within the macrocycle of scytonemide A was hypothesized to be a potential site of attack by the nucleophilic Thr1O^γ^ of the active site; however, the X-ray crystal structure of this natural product in complex with the 20S proteasome to validate the hypothesis has not been reported. Recently, the cyclic hexapeptide baceridin (**42**) was isolated from the culture broth of an epiphytic *Bacillus* strain and underwent total synthesis by the Kalesse group.^119^ Baceridin inhibits all three catalytic sites of the 20S proteasome and inhibits proliferation in several tumor cell lines.

### β-Lactones

**Chart 10:**
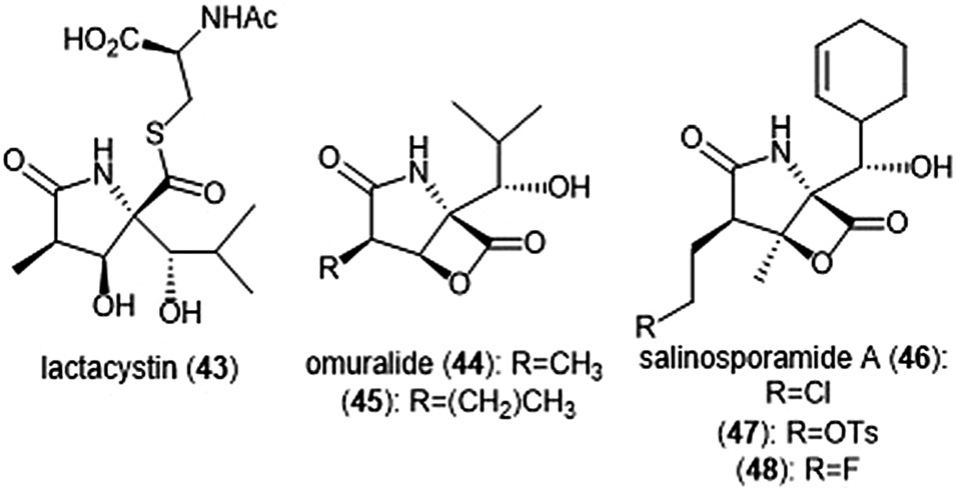
Several natural products and derivatives which contain or form a reactive β-lactone ring and inhibit the proteasome are depicted.

The β-lactone natural products have also been heavily evaluated for their ability to inhibit the proteasome (Chart 10). Lactacystin (**43**) was isolated by Omura *et al.* from *Streptomyces* sp. OM-6519 and displayed biological activity in its initial discovery.^120,121^ In their 1994 study, Fenteany *et al.* researchers further evaluated lactacystin and analogues including omuralide for their biological activity. Results suggested the contribution of either the *N*-acetyl cysteine or β-lactone moieties towards their ability to affect cell cycle progression in Neuro 2A and MG-63 osteosarcoma cells, although the molecular target of these substrates was unidentified at the time.^122^ A subsequent study indicated that lactacystin inhibits the 20S proteasome.^123^ Researchers later determined that rather than acting as an inhibitor towards the 20S proteasome, lactacystin acts as a prodrug of its active β-lactone omuralide (**44**).^124,125^ The first total syntheses of lactacystin and omuralide were achieved by the Corey group, and many total syntheses and formal syntheses have been recorded since.^126–128^

In an SAR study of the omuralide scaffold, the Corey group determined that while the hydroxy-isobutyl substituent at C9 is necessary for potent inhibition of 20S bovine proteasome, alteration of the methyl substituent at C7 with larger or bulky alkyl groups improves potency towards the target.^129^ Research conducted by Soucy *et al.* further complemented these results with derivative **45**, which contains an *n*-propyl group in place of the methyl substituent at C7. Compound **45** as a *K*_obs_/[I] which is greater than two-fold more potent than omuralide (*K*_obs_/[I] = 46 500 M^−1^ s^−1^).^130^ The compact, densely functionalized omuralide scaffold indicated the importance of exploring non-peptidic natural products as potential inhibitors of the 20S proteasome.

Salinosporamide A was first isolated as a product of the ocean sediment-dwelling bacteria *Salinospora* strain CNB-392 and its structure was elucidated by Feling *et al.*^131^ The natural product shares its unique fused γ-lactam–β-lactone bicyclic ring scaffold with that of omuralide. Salinosporamide A was tested for its cytotoxicity against HCT-116 human colon carcinoma cells, displaying an IC_50_ value of 35.1 nM. Due to its structural similarity to omuralide, salinosporamide A (**46**) was also tested for its inhibitory activity towards 20S proteasome; it exhibits potent inhibitory activity towards the ChT-L site (IC_50_: 1.3 nM). Chauhan *et al.* further investigated the bioactivity of salinosporamide A in *in vitro* and *in vivo* studies.^132^ Salinosporamide A inhibits the ChT-L site with an EC_50_ value of 3.5 ± 0.3 nM as well as the C-L and T-L sites with EC_50_ values of 430 ± 34 nM and 28 ± 2 nM, respectively. Salinosporamide A also induced apoptosis in multiple myeloma cells which are resistant towards conventional and Bortezomib therapies.

**Chart 11:**
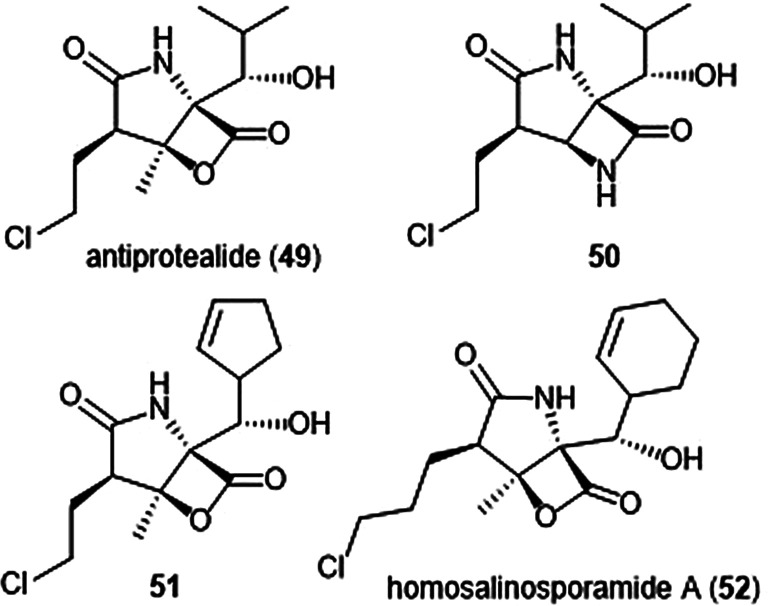
Derivatives of β-lactone natural products are shown.

Only a year after its discovery, the Corey group reported the first total synthesis of salinosporamide A;^133^ the molecule has since become an attractive target for total synthesis, and many syntheses of salinosporamide A have been reported.^134–148^ Exploration of the structure–activity relationship of the salinosporamide scaffold began with Macherla *et al.*,^149^ and has since been a focus of several research groups (Charts 10 and 11). Alteration from the cyclohexenyl ring to other substituted cyclohexanes resulted in lower inhibitory activity towards the 20S proteasome, as well as lower cytotoxicity against multiple myeloma RPMI 8226 cells. Replacement of the chlorine atom with a hydrogen resulted in a 10-fold reduction of its inhibitory activity towards the ChT-L site of the 20S proteasome, as well as a significant reduction of its cytotoxicity towards RPMI 8226 cells. Groll *et al.* later solved the crystal structure of the yeast proteasome core particle (yCP) in complex with salinosporamide A, implicating the importance of the chloroethyl group in the overall inhibitory activity towards the proteasome.^150^ Following transesterification of the β-lactone, the resulting hydroxyl group cyclizes upon the chloro substituent to form a tetrahydrofuran irreversibly.

Manam *et al.* synthesized salinosporamide A analogues which replaced the chloro substituents with functionalities of varying leaving group potentials to further probe the mechanism of inhibition.^151^ All analogues potently inhibit the ChT-L site of the 20S proteasome; tosyl analog **47** displays inhibitory activity towards the ChT-L site of the 20S proteasome with an IC_50_ value of 2.5 ± 0.4 nM. Qualitative dialysis experiments with the analogues indicated that the presence of a leaving group prolongs the duration of inhibition. The researchers also performed kinetics experiments with salinosporamide A to understand the mechanism of its proteasome inhibition, determining that the natural product is a slow tight binding inhibitor which confers its activity through a multi-step inhibition mechanism. Fluorosalinosporamide **48** was later synthesized by Eustáquio and Moore using a combination of genetic engineering and precursor-directed synthesis.^152^ Unlike salinosporamide A, **48** did not undergo displacement of its halogen to afford an irreversible adduct during inhibition, but rather acts as a reversible inhibitor towards the ChT-L activity of the 20S proteasome with two-fold reduced potency relative to salinosporamide A (IC_50_: 1.5 ± 0.05 nM). The ability of the fluorine to participate in hydrophobic interactions with the proteasome was cited as a justification of its inhibitory potency.

The Corey group further explored the salinosporamide A and omuralide scaffolds through the synthesis of a γ-lactam–β-lactone hybrid antiprotealide (**49**).^153^ Antiprotealide—later discovered as a natural product^154^—exhibited 2.5-fold more potent inhibitory activity towards the β5 subunit than omuralide, but was less potent than salinosporamide A. Synthesis of the γ-lactam–β-lactone congener (**50**) of antiprotealide was also successfully achieved by the Corey group. While the analogue displayed slower inactivation towards the 20S proteasome than salinosporamide A and omuralide, its improved stability under physiologic conditions was an advantageous feature for relevant drug development.

McGlinchey *et al.* further demonstrated that bulky groups at the C5 position were necessary for *in vitro* inhibition of the β5-subunit.^155^ Chemical synthesis and metabolic engineering were also used by this group to synthesize several novel salinosporamide derivatives with varying substituents at the P1 position.^156^ The analogue **51**—bearing a cyclopentenyl substituent at the P1 position—exhibited equipotent inhibitory potency towards the chymotrypsin-like site of the proteasome in comparison to salinosporamide A, with an IC_50_ value of 2.2 ± 0.1 nM. Analog **51** also displays increased cytotoxicity against HCT-116 cells.

The length of the chloroalkyl chain at the C2 position of the salinosporamide scaffold is also an important feature with regards to its inhibitory potency. Nguyen *et al.* explored this in their development of an enantioselective route to (—)-salinosporamide A and derivative (—)-homosalinosporamide A.^145^ (—)-Homosalinosporamide A varies from (—)-salinosporamide A only through the length of its C2 sidechain; the compound contains an additional methylene carbon. The researchers proposed that the derivative would behave similarly to salinosporamide A in its mechanism of inhibition towards the human proteasome. (—)-homosalinosporamide A (**52**) displayed similar inhibitory potency towards the ChT-L activity of the 20S proteasome as compared to (—)-salinosporamide A, with an IC_50_ value of 0.7 ± 0.04 nM (*versus* 0.8 ± 0.08 nM). However, X-ray crystallographic evidence of the hCP: (—)-homosalinosporamide A complex illustrated that (—)-homosalinosporamide A does not form a tetrahydropyran during its inhibition of the proteasome.^157^

**Chart 12:**
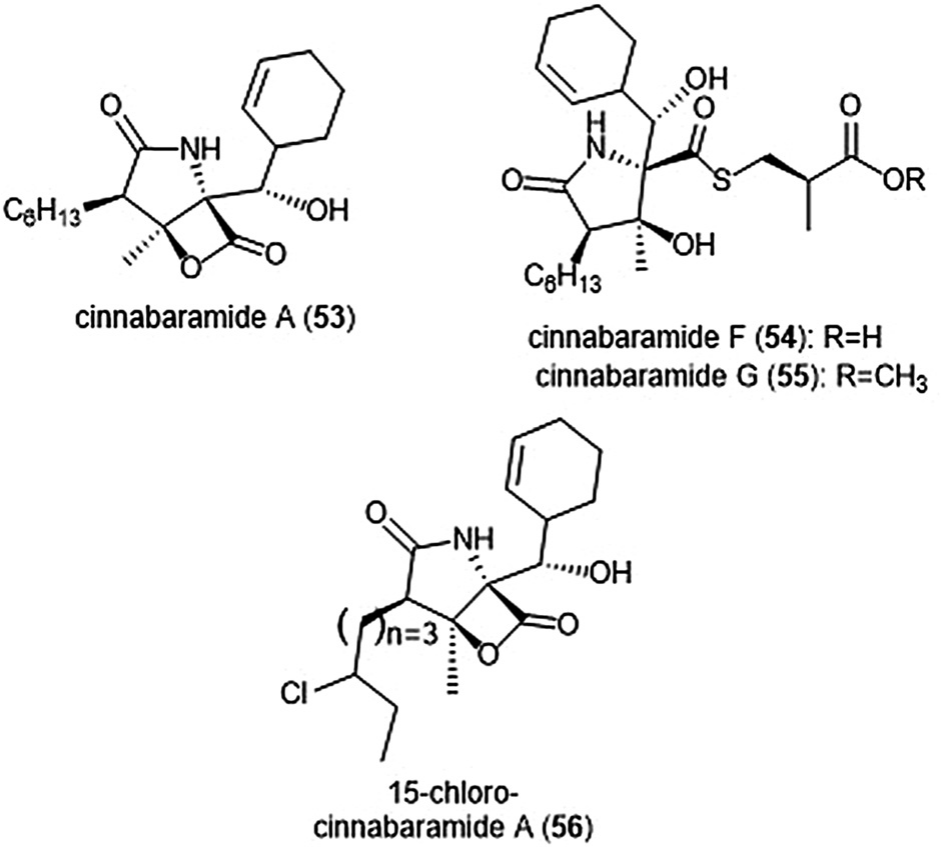
The cinnabaramide natural products and their derivative (**56**) are depicted.

The structurally related cinnabaramides (Chart 12) were first isolated from the terrestrial *Streptomycetes* JS360 in 2007 by Stadler *et al.*^158^*In vitro* evaluation of the inhibitory potency of the cinnabaramides towards the human 20S proteasome mirrored results of previously established inhibitors of similar structure. For example, cinnabaramide A (**53**) (IC_50_: 1 nM) displayed similar potency as salinosporamide A. Cinnabaramide A is also cytotoxic against colon cancer cell line HCT-116 at a level relative to that of salinosporamide A. Cinnabaramides F (**54**) and G (**55**) are believed to act as a prodrug to form the reactive β-lactone in a manner reminiscent to lactacystin; these natural products display potent inhibition towards the human 20S proteasome with IC_50_ values of 6 nM and 0.6 nM, respectively. *rac*-Cinnabaramide A was later synthesized by the Romo group.^137^ In a subsequent study, Rachid *et al.* accessed chlorinated derivatives of cinnabaramides A–D using mutasynthetic methods.^159^ 15-Chlorocinnabaramide A (**56**) exhibits greater inhibitory activity towards the β5 subunit of the 20S proteasome than its parent compound, with an IC_50_ value of 9.3 ± 5.9 nM (IC_50_ value of cinnabaramide A for this assay was 11.9 ± 7.4 nM). Derivative **56** also displays potent cytotoxicity against the HCT-116, RPMI8226, and SW840 cancer cell lines.

**Chart 13:**
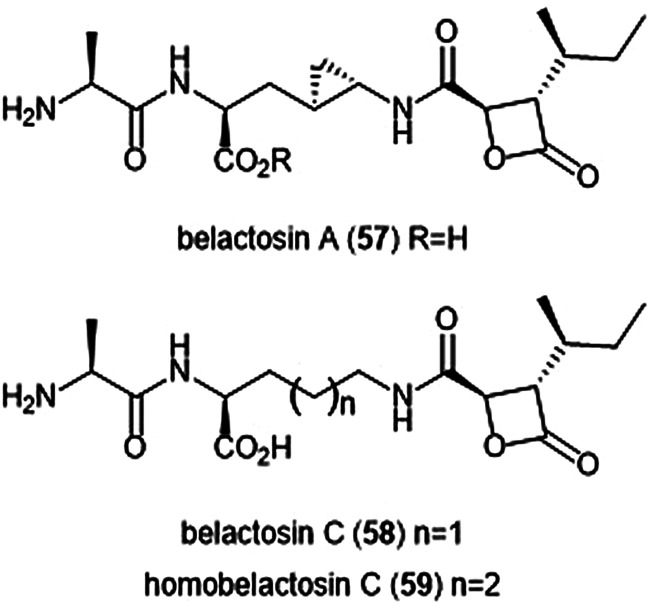
The belactosin inhibitors are depicted. The β-lactone moeity contributes to their ability to inhibit the 20S proteasome.

Belactosins A and C (Chart 13) were first isolated from the culture broth of *Streptomyces* sp. KY11780 by Asai *et al.*^160^ The antiproliferative and antitumor activities of both natural products is attributed to a β-lactone moiety at their C-terminus. Belactosin A contains a cyclopropane ring within its backbone which is absent in that of belactosin C. Belactosin A and C both displayed *in vitro* antiproliferative activity against HeLa S3 cells with IC_50_ values of 51 μM and 200 μM, respectively. Belactosin A further demonstrated the ability to halt cell cycle progression in the G2/M stage in tumor cells. Akai *et al.* reported the molecular mechanism of action of belactosins A (**57**) and C (**58**): both demonstrate nanomolar inhibitory activity towards the ChT-L site of the rabbit 20S proteasome, both displaying IC_50_ values of 0.21 μM.^161^ Optimization of the belactosin A scaffold was further achieved by benzylation of it carboxylic acid. The first total synthesis of belactosin A was completed by Armstrong and Scutt in 2004.^162^ An enantioselective total synthesis of belactosins A, C and its homologue homobelactosin C (**59**) was reported shortly thereafter by Larionov and de Meijere.^163^ X-ray crystallographic evidence based upon the yCP:homobelactosin complex suggested that the β-lactone moiety undergoes nucleophilic attack by the Thr1O^γ^ residue within the catalytic site to form an ester through a covalent, irreversible bond.^164^

**Chart 14:**
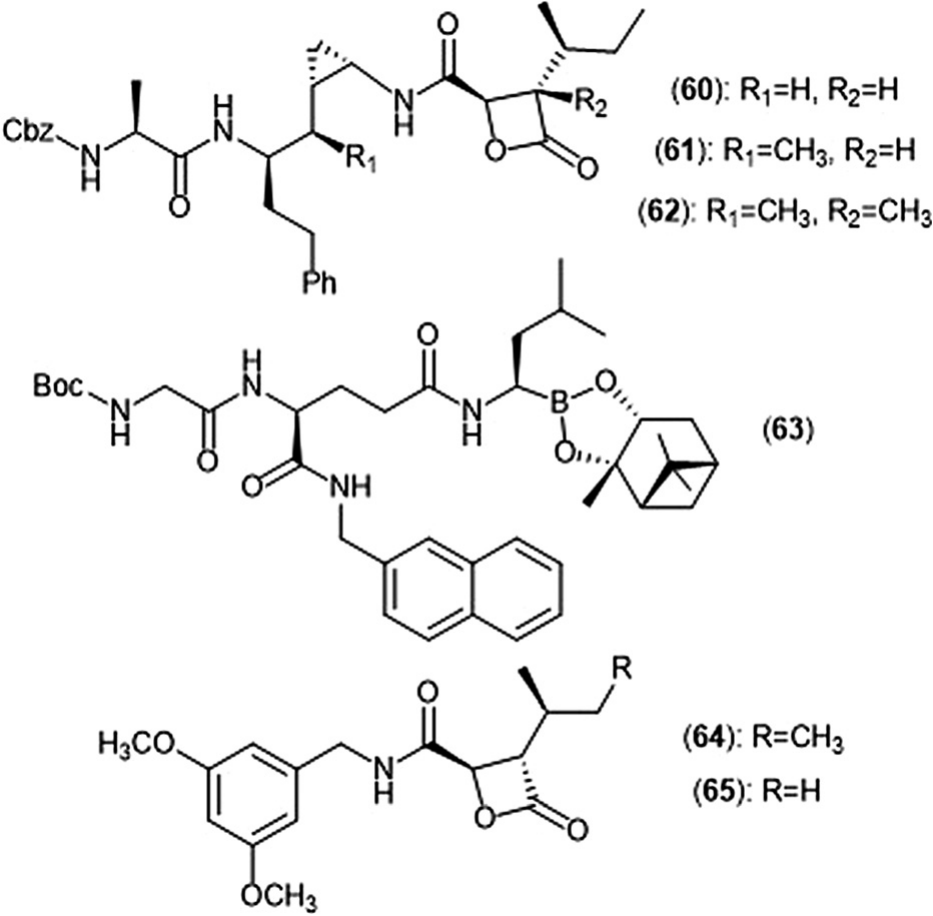
Belactosin-based derivatives which exhibit inhibitory activity towards the proteasome are shown.

Exploration of the stereochemistry surrounding the belactosin A cyclopropyl ring indicated its contribution to biological activity (Chart 14).^165–167^ The (*cis*/L-*anti*) configuration demonstrated promise, as evidenced by compound **60**. Compound **60** selectively inhibits the ChT-L site of the human 20S proteasome with an IC_50_ value of 5.7 ± 1.2 nM, comparable to that of bortezomib. Compound **60** also inhibits the growth of HCT116 cells with an IC_50_ value of 1.82 μM *via* the same mechanism as bortezomib (IC_50_ value: 0.01 μM). The X-ray crystal structure of yCP:**60** complex (2.8 Å), provided researchers with key interactions that contribute to inhibition. Optimization of the transition-state conformation of **60** was subsequently reported.^168^ Installation of a methyl group at carbon C1′ adjacent to the cyclopropyl group led to analog **61**, which is restricted into a predominant *syn* conformation. Derivative **61** exhibited lower inhibitory activity towards the ChT-L site relative to the parent compound with an IC_50_ value of 47 ± 2.9 nM. However, when evaluated for its growth inhibitory activity against several tumor cell lines (Hs-Sultan, KB, and HCT-116), compound **61** exhibits activity similar to its parent compound.

Researchers hypothesized that poor performance of belactosin analogues under biological conditions and in cell studies may be due to chemical or enzymatic hydrolysis of the reactive β-lactone moiety,^168^ and thus sought to improve stability by increasing steric hindrance surrounding the β-lactone ring.^169^ Analogue **62** not only contains an additional methyl group at the α-position of the lactone carbonyl carbon, but also retains the same methyl group at the C1′ carbon adjacent to the cyclopropyl ring to lock it into the *syn* conformation. While analogue **62** displays lower activity towards the ChT-L site of the proteasome as compared to parent compound **60** (IC_50_: 1.3 μM and 0.0057 μM, respectively), **62** exhibited comparable growth inhibitory activity towards HCT-116 cells relative to the established inhibitor (IC_50_: 4.0 μM (**62**); 1.8 μM (**60**)). These results provided a novel belactosin analogue with improved biological stability.

Flexible achiral nonpeptidic analogues with a β-lactone moiety and aromatic tails were synthesized by Kawamura *et al.* using a topology-based scaffold hopping approach.^170^ These synthetically accessible inhibitors represent novel scaffolds with improved drug-likeness in comparison to past belactosin analogues. Simplified nonpeptidic belactosin scaffolds were further explored through synthesis of hybridized bortezomib and epoxomicin analogs.^170,171^ Divergence from the β-lactone moiety of the belactosin scaffold stemmed from an interest to incorporate more stable electrophilic warheads.

Nakamura *et al.* first sought to optimize the belactosin C scaffold in 2009 through the introduction of a boronic acid moiety like that of bortezomib.^172^ Compound **63** was identified as a potent inhibitor of the ChT-L (β5) site of the 20S proteasome (IC_50_: 0.28 ± 0.04 μM) and also exhibited submicromolar growth inhibitory activity against HeLa cells (IC_50_: 0.35 ± 0.02 μM) in an MTT assay. The de Meijere group expanded their previous synthetic endeavors of belactosin scaffolds through the synthesis of novel belactosin C-based proteasome inhibitors.^173^ A subsequent report by the same group elected to divert from the dipeptide scaffold of belactosin C to investigate more easily accessible analogues.^174^ Analogues containing the *N*-(3,5-dimethoxy)benzyl amido side chain displayed especially potent inhibitory activity towards the β5 subunit. This scaffold was later altered to produce a minimal β-lactone scaffold for selective inhibition of either the β5c or β5i of the human 20S proteasome.^175^ Analogue **64** contains a pseudo-isoleucine P1 side chain, whereas **65** contains a smaller pseudo-valine moiety. This difference in size of the P1 side chain resulted in selective inhibition. Compound **64** exhibited preference towards the β5i site (IC_50_: 14.37 nM) over the β5c (IC_50_: 21.35 nM), whereas **65** preferentially inhibits the β5c (IC_50_: 26.87 nM) over the β5i (IC_50_: 83.62 nM).

**Chart 15:**
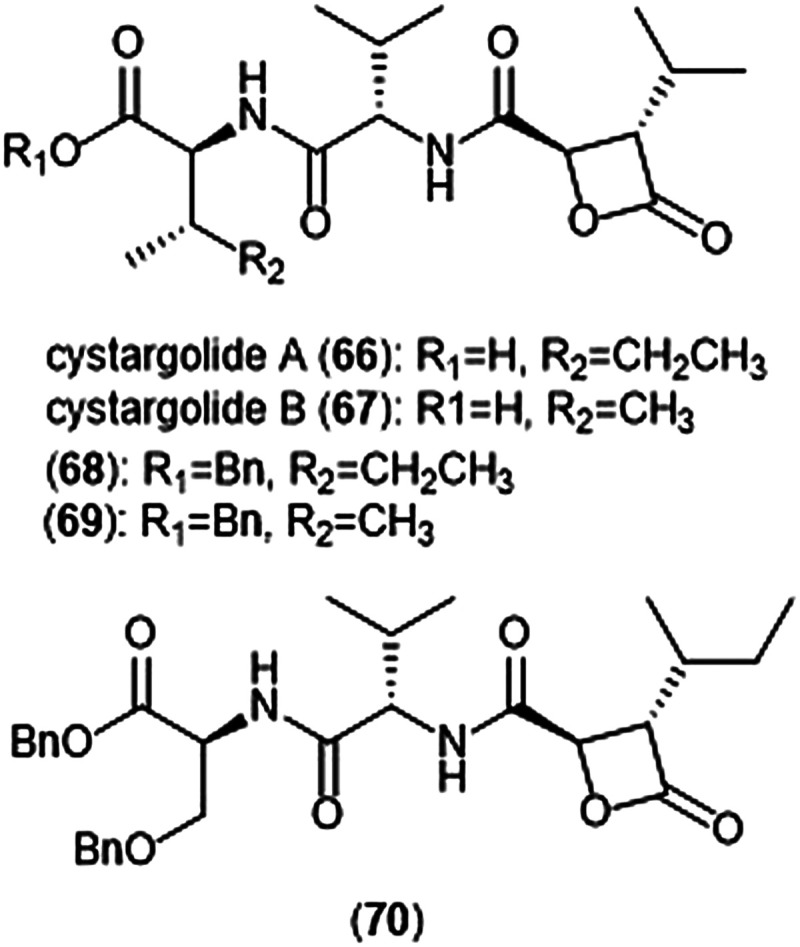
The cystargolide natural products and derivatives are shown.

Another class of β-lactone containing natural products which display intriguing activity towards the proteasome are cystargolides A and B (Chart 15). Originally isolated by the Kerr group in 2015 from the actinomycete *Kitasatospora cystarginea* NRRLB16505, these natural products contain a dipeptide backbone with a β-lactone moiety.^176^ Cystargolides A (**66**) and B (**67**) exhibited inhibition towards the ChT-L site of the 20S proteasome with IC_50_ values of 0.36 ± 0.017 μM and 0.93 ± 0.032 μM, respectively. The total syntheses and absolute stereochemistry of cystargolides A and B were later successfully achieved by Tello-Aburto *et al.*^177^ Wolf *et al.* were also able to access the cystargolides and belactosins using biosynthetic methods. The stereochemistry of these natural products is integral to inhibitory activity: maintaining the (2*R*,3*S*) absolute stereochemistry contributes to potency of inhibitors.^178^ Benzylation of the N-terminus also improved inhibitory potency 100-fold, as evidenced by analogues **68** (IC_50_: 9.2 ± 0.59 nM) and **69** (IC_50_: 9.0 ± 1.4 nM).^177^ Subsequent optimization of the cystargolide scaffold was achieved in 2018: benzyl ether **70** inhibits the hβ5 subunit of Jurkat cell lysate with an IC_50_ value of 3.1 ± 0.2 nM as compared to cystargolide B (IC_50_: 0.90 ± 0.11 μM), and is also cytotoxic against MCF-7 and RPMI-8226 cells.^179^

### α,β-Epoxyketones

**Chart 16:**
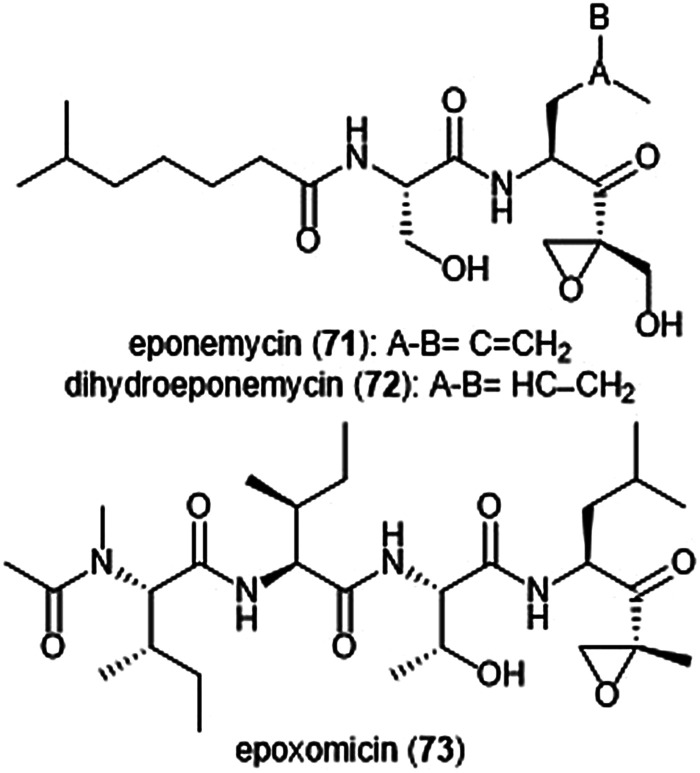
The epoxy-ketone natural products eponemycin, dihydroeponemycin and epoxomicin are depicted. The electrophilic epoxy-ketone moeity within these scaffolds contributres directly to their ability to inhibit the 20S protesome.

The epoxy-ketone containing natural products have become a specific focus to researchers due to their ability to inhibit the 20S proteasome (Chart 16). Eponemycin is a dipeptide natural product which features a lipophilic tail at its N-terminus and an electrophilic α,β-epoxyketone moiety at its C-terminus. The natural product was isolated from the fermentation broth of the soil-dwelling *Streptomyces hygroscopicus* No. P247-71 (ATCC 53709).^180^ Eponemycin (**71**) and its derivative dihydroeponemycin (**72**) exhibit potent *in vitro* toxicity against B16–F10 and HCT-116 cells. The natural product also demonstrated itself as an inhibitor of angiogenesis.^181^ The cellular target of its antitumor activity was later identified by the Crews group as the proteasome with biotinylated eponemycin analogs.^182^ Dihydroeponemycin inhibits the proteasome in a competitive and irreversible manner, with the highest rate of inhibition towards the ChT-L site (*k*_assoc_: 66.4 ± 8.9 M^−1^ s^−1^). Kim *et al.* further explored the structure–activity relationship of dihydroeponemycin and analogues to improve understanding of subunit selectivity within the 20S proteasome.^183^ The ability of dihydroeponemycin to interact with the immunoproteasome was attributed to its lipophilic C-terminus.

The natural product epoxomicin (**73**) was isolated from a soil-dwelling actinomycete no. Q996-17^184^ and is comprised of a tetrapeptide skeleton with a C-terminal α,β-epoxyketone. Epoxomicin exhibited strong *in vitro* cytotoxicities against various cancer cell lines including HCT-116 (IC_50_: 9.0 nM), and additionally exhibits antitumor activity against B16 melanoma. The total synthesis of epoxomicin was first reported by the Crews group in 1999.^185^ Due to previous reports that indicated peptide α,β-epoxyketones as inhibitors of the proteasome,^186^ epoxomicin was evaluated for this activity. Epoxomicin selectively inhibits the chymotrypsin-like site of the 20S proteasome (*k*_assoc_: 35 400 M^−1^ s^−1^). The N-terminal P3 and P4 residues play a major role in the potency of inhibition towards the proteasome.^183^ As compared to dihydroeponemycin, epoxomicin and its analogues display greater inhibitory potency. The X-ray crystal structure of the yCP:epoxomicin complex was solved by Groll *et al.* (2.25 Å resolution),^187^ revealing the basis for its selectivity towards the ChT-L site. The α,β-epoxyketone was initially believed to undergo a reversible nucleophilic addition by the Thr1O^γ^ to produce a hemiacetal; subsequent attack of the epoxide by Thr1N forms a morpholino adduct irreversibly. Recently, the mechanism of proteasome inhibition by epoxyketones was revised based on X-ray crystallographic data by Schrader *et al.*^188^ Instead of forming a morpholino adduct, epoxyketones are believed to react with the Thr1 residue of the catalytic site to form a 1,4-oxepane ring.

**Chart 17:**
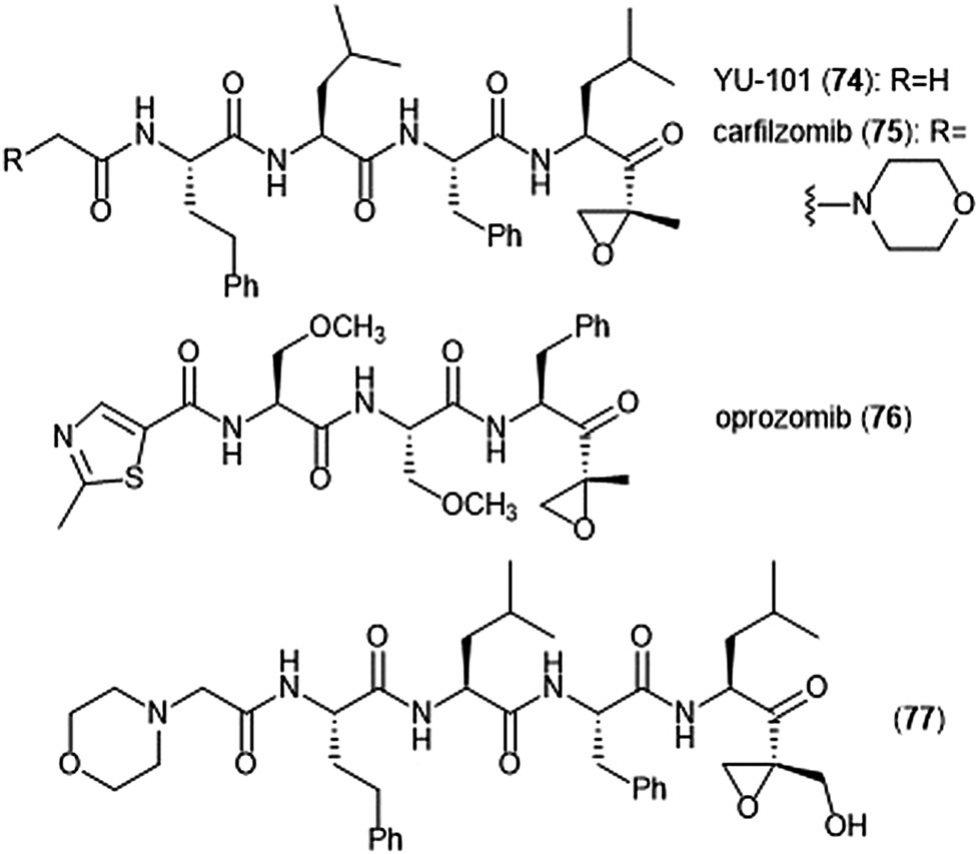
Synthetic inhibitors of the 20S proteasome based upon epoxomicin are shown.

Further optimization of the epoxomicin scaffold has been achieved by several groups (Chart 17). The Crews group set out to improve upon this natural product to access more potent and selective analogues, leading to the development of the tetrapeptide epoxyketone YU-101 (**74**).^189^*In vitro* studies indicate that YU-101 selectively inhibits the ChT-L site of the 20S proteasome with a *k*_assoc_ value of 166 000 (5–12 nM), greater than that of epoxomicin and bortezomib. Further optimization of this analogue led to carfilzomib (Kyprolis®, **75**) by the biotech company Proteolix.^190^ Carfilzomib exhibits selective, potent inhibitory activity towards the ChT-L site of the proteasome, with an IC_50_ value of 6 nM; the compound also performed well in *in vivo* studies. In 2012 carfilzomib was approved by the FDA for the treatment of refractory multiple myeloma;^191^ further alteration of this compound led to the orally available oprozomib (**76**).^192^ Subsequent optimization of the epoxyketone scaffold has focused on developing analogues which can overcome bortezomib and carfilzomib-resistant multiple myeloma. Kim *et al.* recently reported a novel epoxyketone **77**, which not only inhibits the ChT-L activity of proteasome from RPMI8226 cell lysates (IC_50_: 2.1 ± 0.9 nM), but also inhibits proteasome activity (ChT-L) nearly three-fold more potently in RPMI8226-Cfz resistant cells than carfilzomib itself (IC_50_: 106.2 ± 28.9 nM).^193^ Other recent advances of the epoxyketone moeity include the design of selective inhibitors for the β1i,^194,195^ β2i,^196,197^ and β5i subunits.^198^

**Chart 18:**
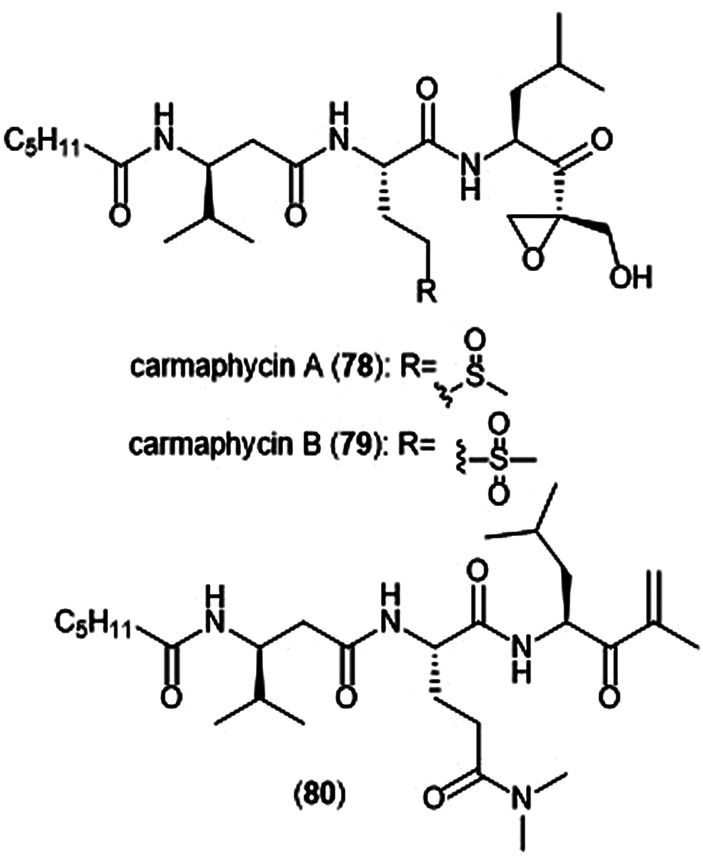
Carmaphycins A and B, and the enone derivative (**80**) are shown.

The epoxyketone-containing natural products carmaphycin A and B were isolated from marine cyanobacterium *Symploca* sp.^199^ These tripeptides contain a lipophilic *N*-acylated tail at their N-terminus and a leucine-derived epoxyketone at their C-terminus. A scalable total synthesis of both carmaphycins was also conducted and both were evaluated for their ability to inhibit the yeast 20S proteasome. Carmaphycins A (**78**) and B (**79**) (Chart 18) exhibited potent inhibitory activity towards the ChT-L site with IC_50_ values of 2.5 ± 0.3 nM and 2.6 ± 0.9 nM, respectively. The natural products also displayed cytotoxic activity towards H-460 and HCT-116 cancer cell lines. Structural investigation implicated the importance of the sulfoxide/sulfone moieties in the methionine-derived residue in the interaction with the target; this interaction represents a distinctive binding mode for the carmaphycins relative to previously discovered α,β-epoxyketone inhibitors. Replacement of the α,β-epoxyketone moiety with an enone led to the identification of analogue **80**, which is a selective nanomolar inhibitor towards the ChT-L site of the 20S yeast proteasome.^200^ Analogue **80** interacts through a unique two-step hydroamination mechanism with the catalytic site to form a morpholine adduct. Most recently, carmaphycin-based proteasome inhibitors have been designed for use as antibody drug conjugates as potential cancer treatments.^201^

**Chart 19:**
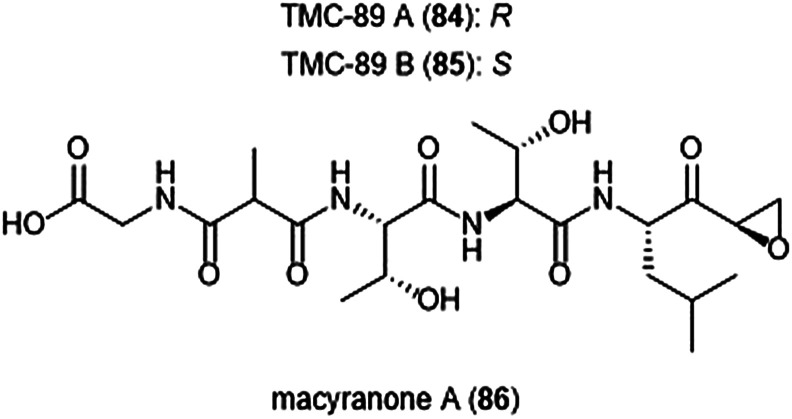
Several epoxy-ketone containing peptide natural products have been identified as inhibitors of the 20S proteasome.

Several other epoxyketone natural products were also isolated and evaluated for their ability to inhibit the proteasome (Chart 19). TMC-86A (**81**) and B (**82**) are isolated from the fermentation broth of *Streptomyces* sp. TC 1084.^202,203^ Both display similar inhibitory potency towards the ChT-L site with IC_50_ values of 5.1 μM and 1.1 μM, respectively. TMC-86A and B also exhibited nanomolar cytotoxicity against various tumor cell lines. TMC-96 (**83**) was isolated from the fermentation broth of *Saccharothrix* sp. TC 1094,^202,203^ and contains a branched *N*-acylated terminus and a leucine-derived α,β-epoxyketone moiety similar to dihydroeponemycin. TMC-96 exhibits inhibitory activity towards the ChT-L and C-L sites of the 20S proteasome (IC_50_: 2.9 μM and 3.5 μM, respectively), and additionally displays nanomolar cytotoxic activity against a variety of tumor cell lines. TMC-89A (**84**) and B (**85**) are α,β-epoxyketone-containing tripeptides which were isolated from the fermentation broth of *Streptomyces* sp. TC 1087 by Koguchi *et al.* in their search for new proteasome inhibitors of bacterial origin.^204^ When tested for their proteasome inhibitory activity, both displayed equipotent micromolar activity towards the ChT-L site of the 20S proteasome, with IC_50_: 1.1 μM. TMC-89A and B also exhibit slight selectivity towards the T-L site of the 20S proteasome, with IC_50_ values of 390 nM and 510 nM, respectively. *In vitro* cytotoxicity studies reveal that in comparison to many other α,β-epoxyketones, TMC-89A and B are not remarkably cytotoxic against tumor cell lines. Subsequent optimization of these natural products has not been reported but may benefit from the addition of N-terminal lipophilic residues to improve cell permeability.

The macyranones are a group of six linear peptides which were produced by the myxobacteria *Cystobacter fuscus* MCy9118.^205^ Macryanone A (**86**) contains an α,β-epoxyketone moiety which made it an intriguing compound to test for proteasome inhibition. Macyranone A inhibits the ChT-L activity of the yCP (IC_50_: 5.9 nM) and the human constitutive proteasome and immunoproteasome, with IC_50_ values of 21 nM and 15 nM, respectively. X-ray crystallographic analysis of the yCP: macyranone complex (2.8 Å resolution) revealed that it reacts irreversibly with the catalytic sites of the proteasome in a similar mechanism to epoxomicin. Despite its promising performance in *in vitro* enzymatic testing, macyranone A displayed poor cytotoxicity against mammalian cell lines including HCT-116, THP-1 and HL-60 cancer cell lines.

**Chart 20:**
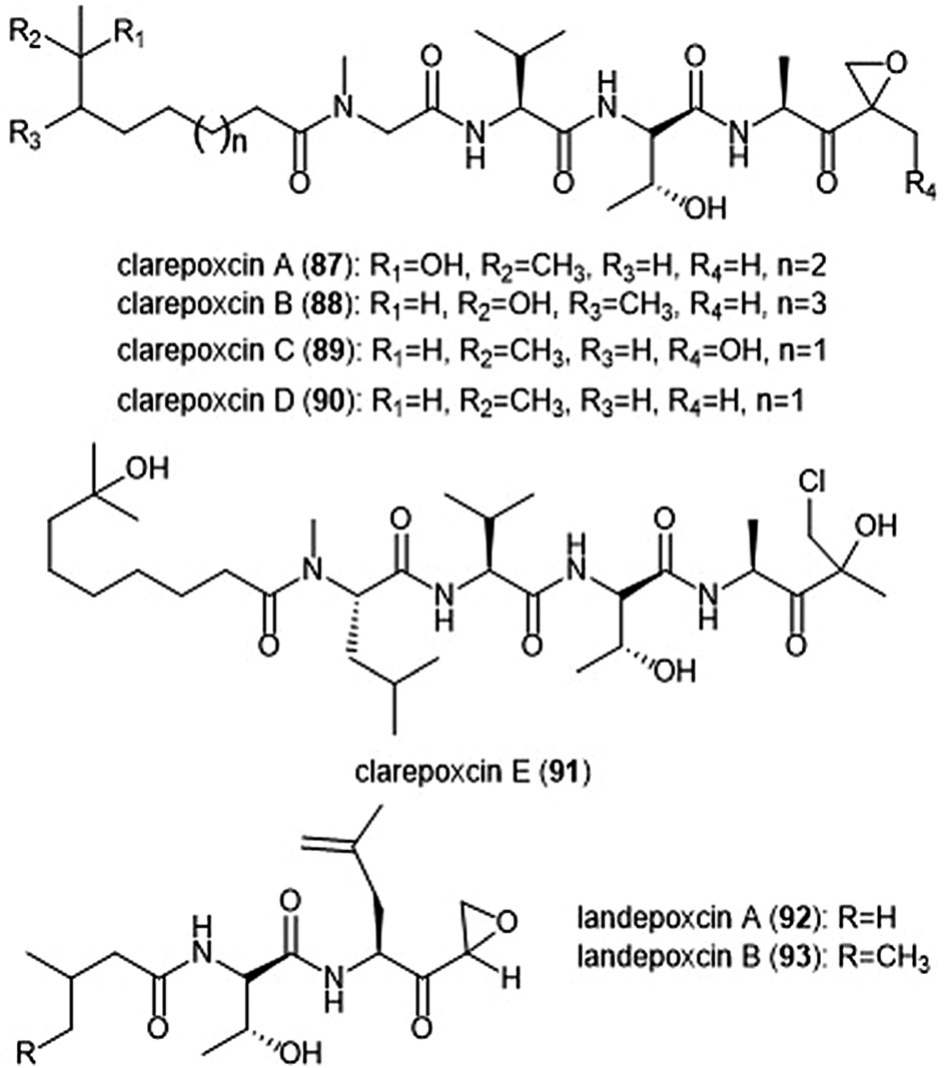
The recently identified epoxy-ketone containing proteasome inhibitors clarepoxcins and landepoxcins are shown. Clarepoxcin E is believed to act as a prodrug of an epoxy ketone in cell studies.

The most recently discovered epoxyketone natural product inhibitors were reported by Owen *et al.* The group utilized molecular evolutionary analyses of complex metagenomes to target and isolate the previously unidentified clarepoxcin and landepoxcin natural products (Chart 20).^206^ Clarepoxcins A–E (**87–91**) were produced by *S. albus:AR456*, while landepoxcins A (**92**) and B (**93**) were produced by *S. albus:AR412.* Clarepoxcins A–D are potent low nanomolar inhibitors of the human 20S proteasome, with an IC_50_ ∼ 6.9–15.1 nM (ChT-L). The clarepoxcins also exhibited potent cytotoxic activity against HCT-116 cells. While clarepoxcin E did not display inhibitory activity towards the 20S human proteasome, it did exhibit potent cytotoxicity against HCT-116. The authors proposed that in cells, clarepoxcin E acts as a prodrug to form the reactive α,β-epoxyketone moiety. Landepoxcins A and B displayed higher nanomolar inhibitory activity towards the human 20S proteasome, however landepoxcin A did exhibit impressive cytotoxicity against HCT-116 cells.

### Terpenoids

**Chart 21:**
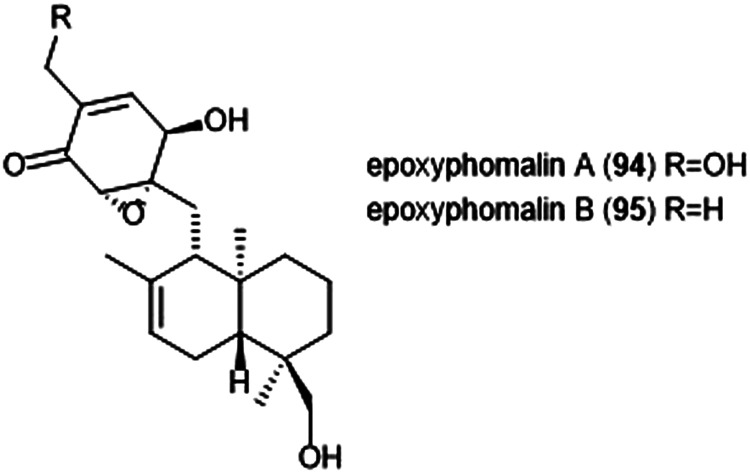
The proteasome inhibitors epoxyphomalin A and B are shown.

Terpenoids represent a vast class of natural products that have been reported for their biological activities. For example, the agosterols were evaluated by the Tsukamoto group for their ability to inhibit the proteasome. Among the many tested, agosterol C demonstrated the most potent activity against the ChT-L site, with an IC_50_ value of 19.8 μM.^207^ Epoxyphomalins A and B were first isolated as products of the marine-derived fungus *Phoma* sp. by Mohamed *et al.*^208^ Epoxyphomalin A displayed cytotoxicity towards several human tumor cell lines. The intracellular target of epoxyphomalins A and B was later identified as the 20S proteasome;^209^ both epoxyphomalins A (**94**) and B (**95**) exhibit dose-dependent inhibition of the 20S human proteasome subunits (Chart 21). Epoxyphomalin A demonstrated equipotent inhibition against all three catalytic sites, whereas epoxyphomalin B preferentially inhibits the ChT-L site.

**Chart 22:**
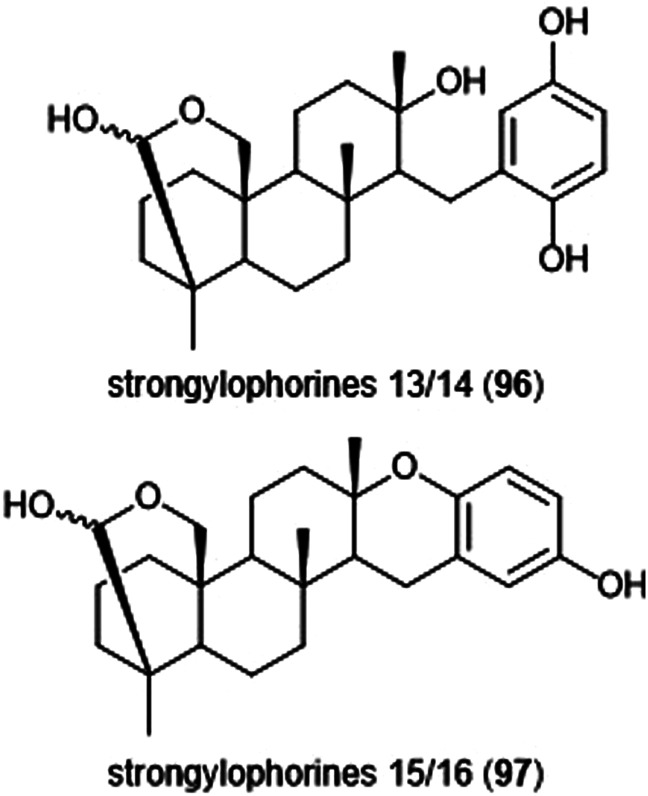
The hemiacetal moieties in strongylophorines 13/14 and 15/16 contribute to their ability to inhibit the 20S proteasome.

Noda *et al.* isolated a variety of stronglyphorines from marine sponge *Petrosia corticata* in 2015.^210^ The structure–activity relationship of the strongylophorines with their inhibitory activity revealed that the presence of a hemiacetal in addition to a hydroquinone moiety contributes significantly towards the potency of the proteasome inhibitors. Strongylophorines 13/14 (**96**) and strongylophorines 15/16 (**97**) exhibited the most potent inhibitory activity towards the ChT-L site of the 20S proteasome with an IC_50_ of 2.1 μM and 3.6 μM, respectively (Chart 22).

**Chart 23:**
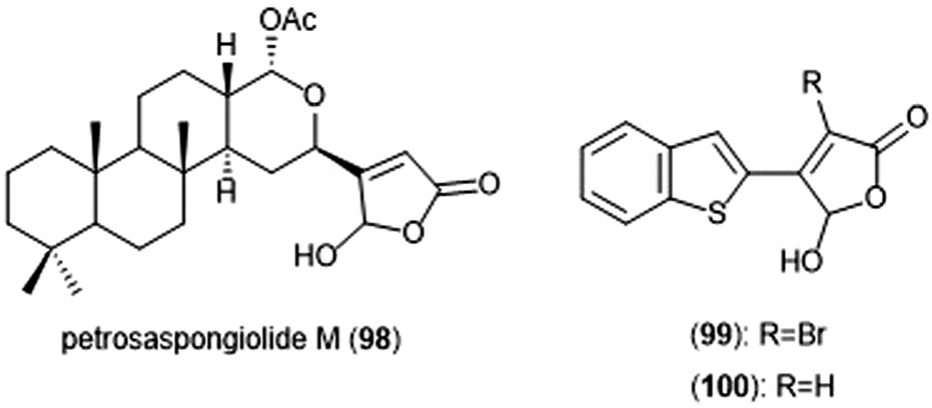
Petrosaspongiolide M and its simplified analogues are depicted; all three inhibit the activity of the proteasome.

Petrosaspongiolide M (**98**) was isolated from the New Caledonian marine sponge *Petrosaspongi nigra* among the other petrosaspongiolides A–R. The petrosaspongiolides are sesterterpenes all containing a cheilantane skeleton, and were first identified as inhibitors of the preparation of phospholipase A_2_.^211^ Petrosaspongiolide M was later identified by Margarucci *et al.* as a proteasome inhibitor using chemical proteomics.^212^ The natural product inhibited the C-L and ChT-L activity of the proteasome in a cell-based assay with IC_50_ values of 0.85 ± 0.15 μM and 0.64 ± 0.15 μM, respectively. Petrosaspongiolide M was later reported to exhibit potent inhibitory activity towards the ChT-L and C-L activities of the proteasome with submicromolar values in an *in vitro* fluorometric enzyme assay.^213^ Optimization led to analogues **99** and **100**, which both feature a benzothiophen-2-yl substituent at the C4 position of the butenolide ring. Analogue **99** exhibited potent inhibitory activity against the ChT-L and C-L site of the 20S proteasome, with IC_50_ values of 0.06 ± 0.009 and 0.22 ± 0.03 nM, respectively. The desbrominated analogue **100** also displayed potent inhibitory activity towards the ChT-L and C-L sites, with IC_50_ values of 0.07 ± 0.01 nM for both sites (Chart 23).

**Chart 24:**
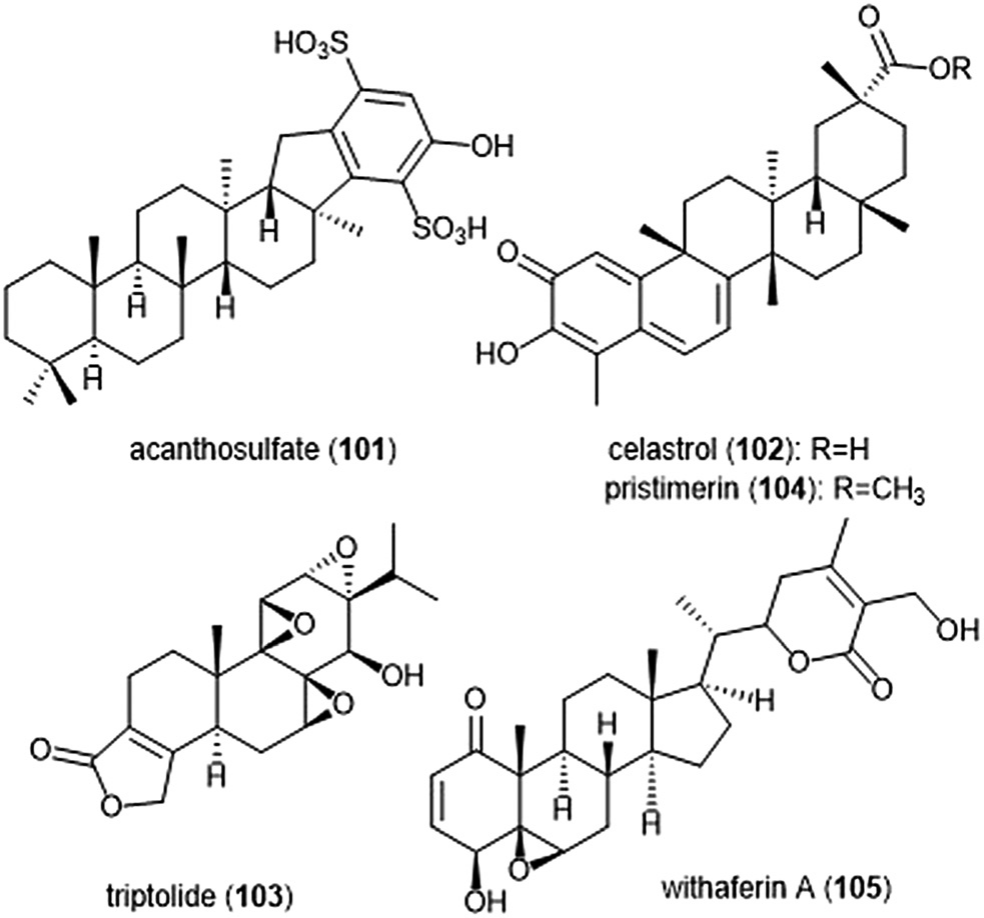
Various terpenoids which have been identified as proteasome inhibitors are depicted.

Several other terpenoid natural products have also been identified as inhibitors of the 20S proteasome. The merosesterterpene acanthosulfate (**101**) was identified by West and Faulkner as a metabolite of the marine sponge *Acanthodendrilla* sp. in 2008.^214^ Acanthosulfate (Chart 24) exhibited inhibition towards the proteasome (IC_50_: 4.5 μM) but lacked selectivity and potency when tested for activity against the BMS Oncology Diverse Cell Panel (ODCA).

Celastrol (**102**) is a quinone methide triterpene which is extracted from the “Thunder God Vine” (*Tripterygium wilfordii* Hook F.) and has been recognized for its biological activity. Dou *et al.* discovered the inhibitory activity of celastrol towards the 20S proteasome in 2006.^215^ Celastrol selectively inhibits the ChT-L activity of the proteasome with an IC_50_ value of 2.5 μM. The compound also demonstrated the ability to inhibit the proteasome of prostate cancer cells in a cell-based assay at 1–5 μM. Celastrol further demonstrated antitumor activity in an *in vivo* study. Researchers reported that treatment of PC-3 containing mice with the natural product resulted in tumor reduction. Because celastrol has many intracellular targets,^216^ focus on optimization of the celastrol scaffold has involved improving its solubility and physiological properties. Recently, celastrol has been reported as cytotoxic against human multiple myeloma cells.^217^ Further investigation of compounds from the extract of the “Thunder God Vine” (*Tripterygium wilfordii* F. Hook) focused on the diterpene tri-epoxide lactone triptolide (**103**). Originally discovered in 1972, researchers reported its antileukemic activity thus providing impetus for further biological evaluaton.^218,219^ Although triptolide does not inhibit the ChT-L site of purified 20S proteasome, it was able to inhibit proteasomal activity within cancer cells. This interesting result suggests that triptolide acts as a prodrug, and one of its metabolites—perhaps an oxidized ketone—acts instead as the biologically active molecule.

Tiedemann *et al.* further demonstrated the inhibitory potential of the celastrol scaffold through the investigation of its natural product relative pristimerin and the mechanisms of its biological activity.^220^ Pristimerin (**104**) contains an identical scaffold to that of celastrol except for the carboxylic acid moiety, which is instead replaced by a methyl ester. Pristimerin selectively inhibits the ChT-L activity of the 20S proteasome with an EC_50_ value of less than 125 nM. Researchers further indicated in a cell-based experiment that pristimerin suppresses NF-KB activity through proteasome dependent and independent pathways. Pristimerin also demonstrated promising activities in cytotoxicity and *in vivo* studies. The steroidal lactone withaferin A (**105**) is a natural product which was isolated from the Indian Winter Cherry *Withania somnifera* Dunal, a plant which is popular for its use in Ayurvedic medicine.^221^ The steroid contains two conjugated ketones and bears structural similarity to celastrol. Withaferin A had previously been recognized for its various biological activities, though the mechanism of action by the natural product had not been elucidated. Dou *et al.* identified the 20S proteasome as an intracellular target of withaferin A.^222^ Withaferin A inhibits the ChT-L activity of the 20S proteasome with an IC_50_ value of 4.5 μM and also exhibits proteasomal inhibition within PC-3 cells. The natural product also performed well in *in vivo* tumor studies conducted on mice. *In silico* docking studies in addition to kinetic studies suggested that the conjugated ketone moieties of withaferin A may interact covalently with the Thr1O^γ^ within the β5 active site to confer inhibition of the 20S proteasome.

**Chart 25:**
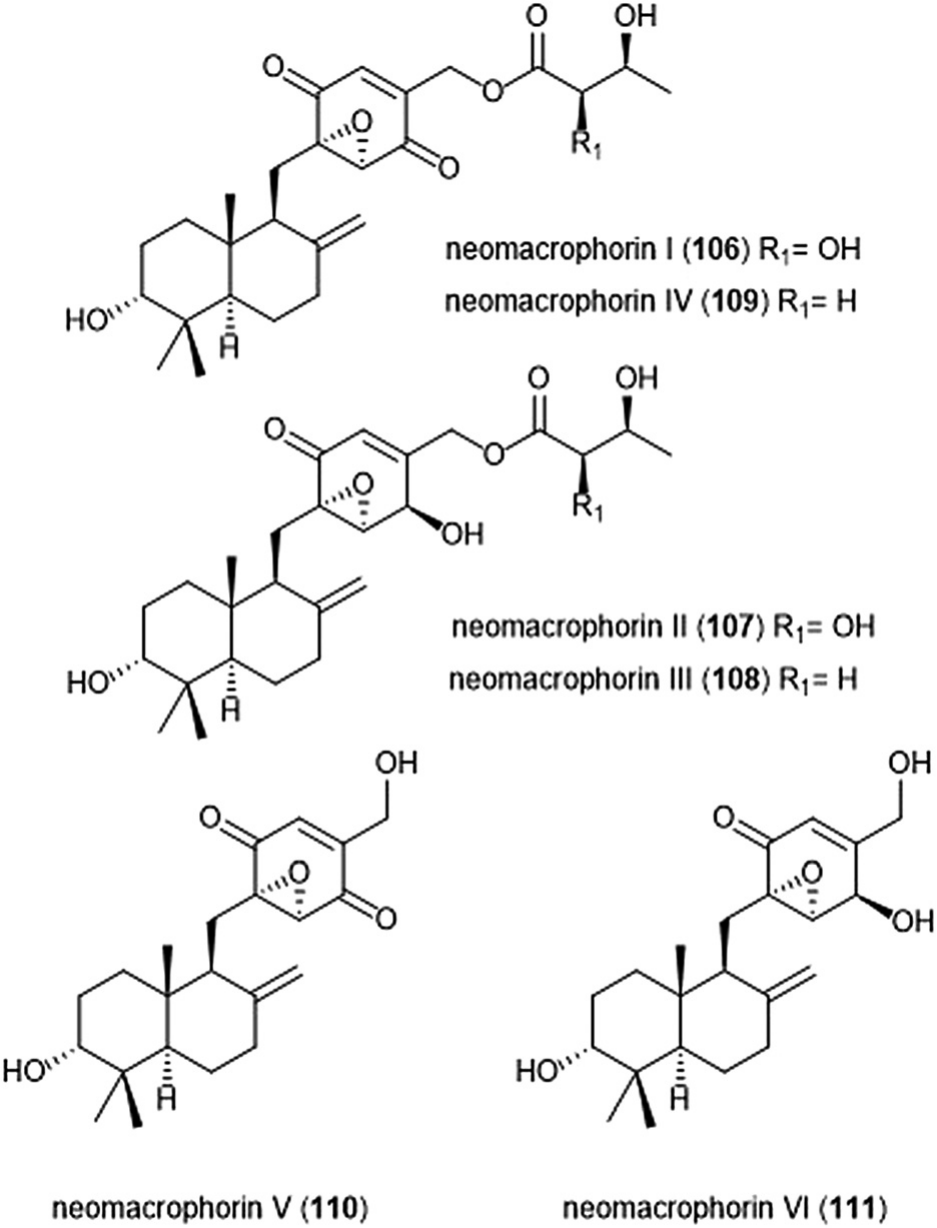
The neomacrophorins I–VI are depicted.

The neomacrophorins (Chart 25) were identified as products of soil-dwelling *Trichoderma* sp. 1212-03, and have recently been evaluated for their biological activities.^223–225^ Neomacrophorin I demonstrated cytotoxicity against human colorectal cancer COLO 201 cells, though its mode of action for this cytotoxicity was initially unclear. An MTT assay of the neomacrophorins with promyelocytic leukemia HL60 cells revealed that neomacrophorins I–VI (**106–111**) inhibit growth of the HL60 cells. The molecules were also responsible for inducing apoptotic cell death in HL60 cells, and further demonstrated *in vitro* inhibitory activity towards the 20S proteasome. Neomacrophorins I and IV demonstrated the most potent inhibitory activity towards the ChT-L site as compared to the other neomacrophorins, with IC_50_ values of 5.7 ± 1.0 and 5.3 ± 1.2 μM, respectively. The quinone moiety was deemed an important feature for the inhibitory proteasome activity and cytotoxicity.

### Alkaloids

**Chart 26:**
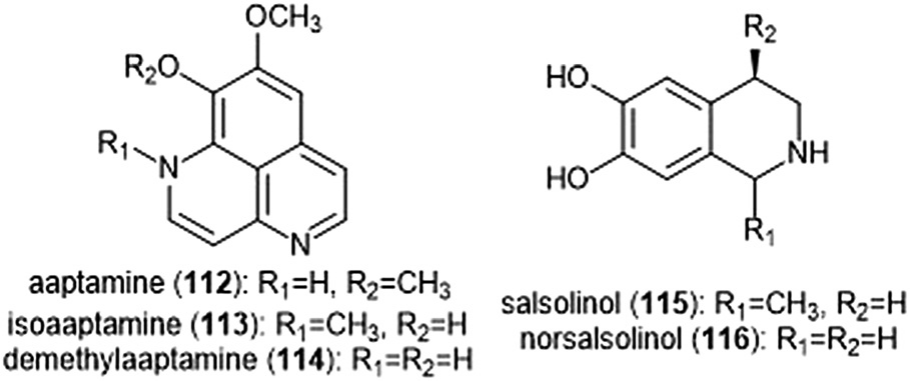
The aaptamines and salsolinols all inhibit the 20S proteasome.

Various alkaloids have also been identified as inhibitors of the proteasome. Tsukamoto *et al.* isolated the aaptamine natural products (Chart 26) from the marine sponge *Aaptos suberitoides* as part of their efforts to identify novel proteasome inhibitors from marine invertebrate extracts and marine-derived fungi cultures. Aaptamine (**112**), isoaaptamine (**113**) and demethylaaptamine (**114**) inhibit the ChT-L and C-L sites for both rat and human 20S proteasomes (IC_50_: 7.0–20.2 μM). When tested for cytotoxicity against HeLa cells, the aaptamines did not exhibit a correlation between cytotoxicity and proteasome inhibition.

The tetrahydroisoquinoline salsolinol alkaloids were later isolated from the marine sponge *Xestospongia cf. vansoesti* and evaluated for their proteasome inhibitory activity.^226^ Salsolinol (**115**) and its derivative norsalsolinol (**116**) inhibit the ChT-L site with IC_50_ values of 279.0 and 193.7 μM, respectively. Cytotoxicity studies indicated that salsolinol is cytotoxic against several cell lines; norsalsolinol displayed cytotoxicity against HeLa cells (IC_50_: 42.4 μM) but was not tested against other cell lines.

**Chart 27:**
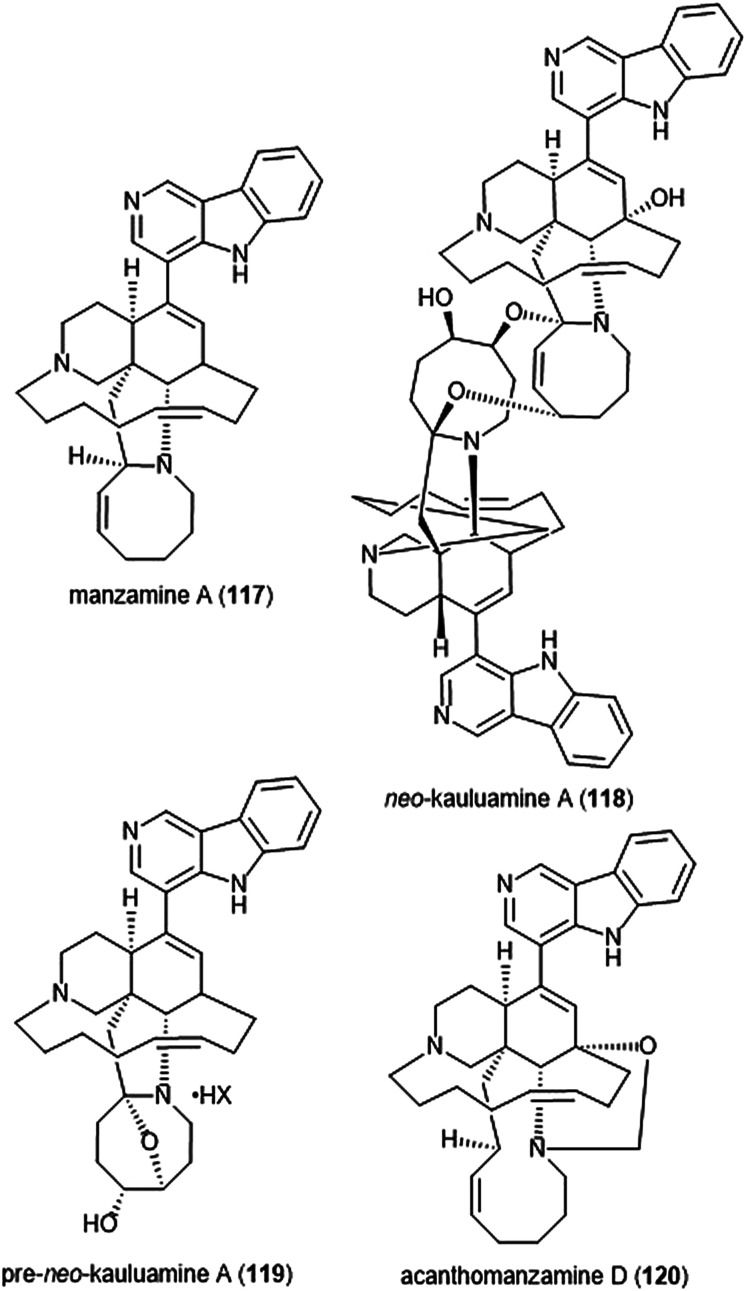
Various manzamines have been identified for their ability to inhibit the 20S proteasome. The eight-membered ring has been credited as an integral contributor to inhibition.

Specific manzamines have been identified as inhibitors of the 20S proteasome by Tsukamoto *et al.* (Chart 27).^227,228^ Following their isolation from the marine sponge *Acanthostrongylophora ingens* and structural identification, several were evaluated for their cytotoxicity and proteasome inhibitory activity. Manzamine A (**117**), *neo*-kauluamine (**118**) and pre-*neo*-kauluamine (**119**) exhibit potent inhibitory activity towards the ChT-L site of the 20S proteasome with IC_50_ values of 2.0, 0.13 and 0.34 μM, respectively. Acanthomanzamine D (**120**) also exhibits nanomolar inhibitory activity towards the chymotrypsin-like site of the 20S proteasome (IC_50_: 630 nM), albeit with less potency than the kauluamines. The presence of the eight-membered ring in addition to the β-carboline appeared to be necessary for proteasome inhibition.

**Chart 28:**
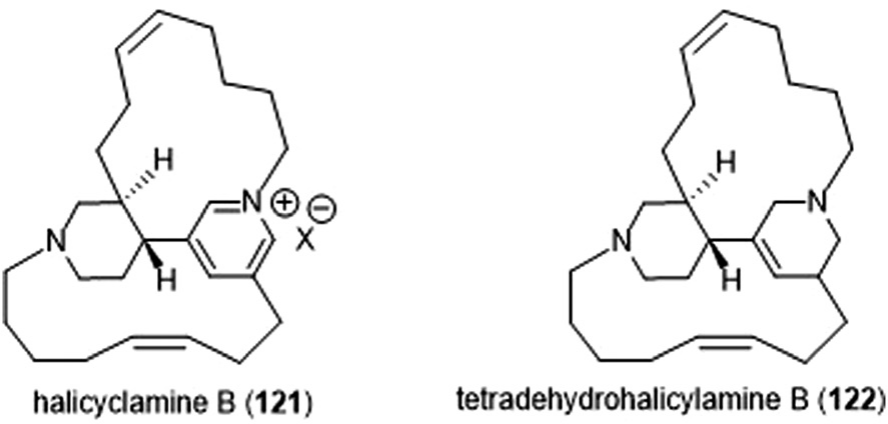
Halicylamine B and tetradehydrohalicyclamine B are shown.

The Tsukamoto group also isolated two halicyclamines from *Acanthostrongylophora ingens* and evaluated them for their biological activity (Chart 28).^229^ Halicyclamine B (**121**) and tetradehydrohalicylamine B (**122**) differ only in their level of unsaturation; whereas the latter contains a pyridinium ring, the former contains a substituted tetrahydropyridine ring. The halicyclamines were tested for cytotoxic activities against HeLa cells which showed that tetradehydrohalicyclamine B exhibited low micromolar cytotoxicity (IC_50_: 12 μM), whereas halicyclamine B is not cytotoxic. Halicyclamine B and tetradehydrohalicyclamine B demonstrated inhibitory activity towards both the cCP and iCP.

**Chart 29:**
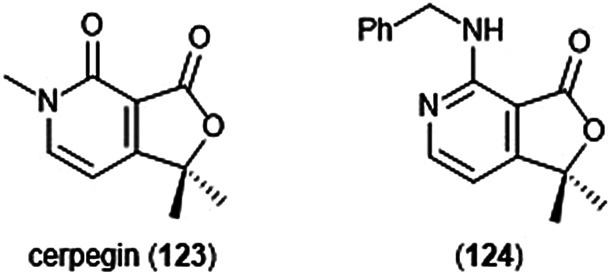
The cerpegin scaffold has also been optimized for proteasome inhibition. Depicted are the natural product and its optimized benzylamino analog (**124**).

Cerpegin (**123**) and its analogs (Chart 29) have been investigated as novel proteasome inhibitors in recent years. Cerpegin itself exhibits selective micromolar inhibition against the C-L activity of the 20S proteasome, with an IC_50_ value of 10.4 ± 0.5 μM.^230^ Optimization of the N^5^ position led to the identification of selective micromolar (IC_50_: ∼5 μM) inhibitors of the caspase-like activity of the 20S proteasome*. In silico* docking suggested an interaction of the N^5^ substituents with a Tyr residue (Tyr114, β2 subunit) for their selectivity. Introduction of a large, flexible hydrophobic residue at the C1 position led to the discovery of sixteen derivatives with micromolar (IC_50_: 2–5 μM) activity towards the β1 subunit.^231^*In silico* docking indicated that these hydrophobic moieties bind within the primed substrate binding channel of the β1 active site to confer selectivity. Replacement of the carbonyl at C4 with a benzylamino moiety further improved the inhibitory activity of the scaffold to lead to β1-selective nanomolar inhibitor (**124**) (IC_50_: 600 nM).^232^

**Chart 30:**
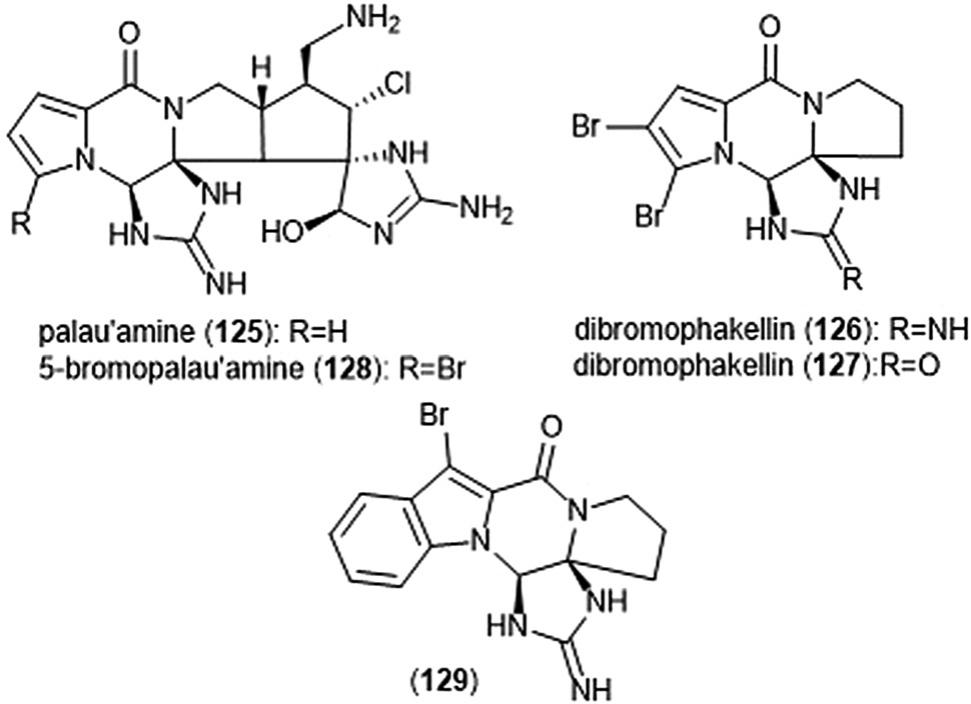
Various pyrrole–imidazole alkaloids and analogs have been identified as inhibitors of the 20S proteasome; a selection are displayed.

Pyrrole–imidazole alkaloids have also been investigated for their ability to inhibit the proteasome (Chart 30). Pyrrole–imidazole alkaloids encompass a large group of heterocyclic natural products isolated from marine sponges.^233^ Perhaps one of the most famous pyrrole–imidazole alkaloids is palau ‘amine (**125**). Discovered by Scheuer *et al.* in 1993,^234^ researchers reported that palau ‘amine displayed cytotoxic activity. The first total synthesis of (±)-palau’amine was achieved by the Baran group in 2010,^235^ followed by an enantioselective synthesis of (—)-palau’amine shortly thereafter.^236^ Due to its reported cytotoxicity, palau’amine and its relatives (±)-dibromophakellin (**126**) and (±)-dibromophakellstatin (**127**) were evaluated as inhibitors of the ChT-L activity of the cCP and iCP.^237^ (—)-Palau’amine displayed 2-fold improved inhibitory activity towards the constitutive human 20S proteasome relative to its racemate (IC_50_: 2.5 (±0.7) *vs.* 5.5 (±1.5) μM, respectively), indicating that the (—) enantiomer is responsible for inhibitory activity towards the proteasome. The presence of a cyclic urea or guanidine ring is integral to inhibitory activity. Researchers synthesized several indole analogs^238^ as potential inhibitors based on the (±)-dibromophakellin scaffold using the same strategy as Hewlett and Tepe in their total synthesis.^239^ (±)-Indolophakellin analog **129** exhibited potent and specific inhibitory activity towards the β5c of the 20S proteasome (IC_50_: 3.5 ± 0.7 μM) at a comparable potency to (±)-palau’amine. An X-ray crystal structure of the yCP:**129** complex (2.5 Å resolution) revealed that the analogue confers its inhibition through solely non-covalent interactions with the S3 subpocket of the β5 subunit, including H-bonding and halogen bonding interactions. Since this study, newly discovered pyrrole–imidazole alkaloids have frequently been tested for their proteasomal inhibitory activity. For example, 5-bromopalau’amine (**128**) was recently isolated among several bromopyrrole alkaloids from the *Dictyonella* sp. marine sponge and exhibited inhibition towards the ChT-L site of the 20S proteasome (IC_50_: 9.2 ± 3.2 μM).^240^

**Chart 31:**
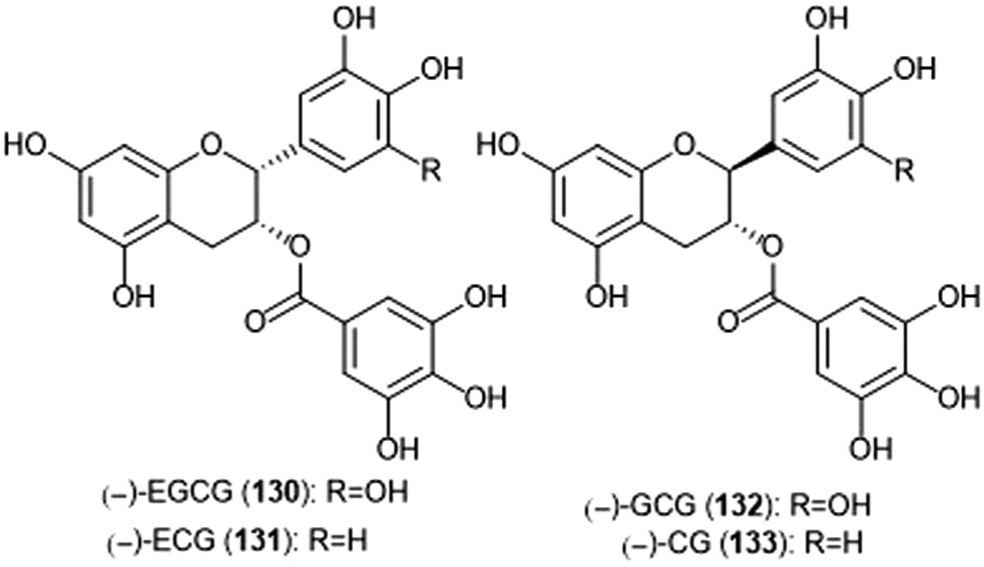
Gallate-containing tea polyphenols which inhibit the 20S proteasome are shown.

### Flavonoids

Many flavonoids have been isolated and evaluated for their ability to inhibit the 20S proteasome based upon their intriguing bioactivities. The green tea-derived catechin polyphenols have been among these flavonoids of interest, and include epigallocatechin-3-gallate ((—)-EGCG), epicatechin ((—)-EC), epigallocatechin ((—)-EGC), gallocatechin-gallate ((—)-GCG), and catechin gallate. The ester moiety present in of some of these flavonoids was thought to contribute to proteasome inhibition, as the ester-containing catechins (—)-EGCG (**130**), (—)-ECG (**131**), (—)-GCG (**132**) and (—)-CG (**133**)(Chart 31) inhibit the chymotrypsin-like activity of the 20S proteasome in a range of IC_50_ values 86–194 nM, (—)-EGCG being the most potent among them.^241^ Catechins lacking the ester moiety do not display this same inhibitory potency. Subsequent cell-based studies using (—)-EGCG demonstrated the compound's ability to inhibit the 26S proteasome in Jurkat T cells; 10 μM (—)-EGCG inhibited ∼70% of the proteasomal ChT-L activity. EGCG also inhibits the ChT-L activity in breast (MCF-7) and prostate (PC-3 and LNCaP) cancer cells. Enantiomeric analogues of natural catechins, (+)-EGCG and (+)-GCG inhibit the ChT-L activity of the proteasome in both *in vitro* and *in vivo* studies to a similar potency of the natural catechins. However, global protection of the hydroxy groups of (+)-EGCG with benzyl moieties renders the compound inactive, implicating that the presence of at least one hydroxyl group is necessary for inhibitory activity.^242^ The mechanism of proteasome inhibition by catechins was later postulated by Smith *et al.*^243^ (—)-EGCG irreversibly inhibits the ChT-L activity of the 20S proteasome in a time-dependent manner, indicative of covalent bond formation within the active site. Docking studies carried out using AutoDock indicated this potential interaction. When docked with the ChT-L site, the ester carbonyl of (—)-EGCG is within 3.18 Å of Thr1O^γ^ for nucleophilic attack. The hydrophobic A ring of (—)-EGCG also sits within the S1 subpocket of the ChT-L site to confer selectivity between subunits. Due to instability of (—)-EGCG under biological conditions, subsequent studies of the tea polyphenols for proteasome inhibition focused upon improving analogue stability under physiologically-relevant conditions. Several studies by the Dou group have been conducted to generate bioactive analogues of (—)-EGCG: examples of these include peracetate esters^244,245^ and fluorinated analogues.^246^

**Chart 32:**
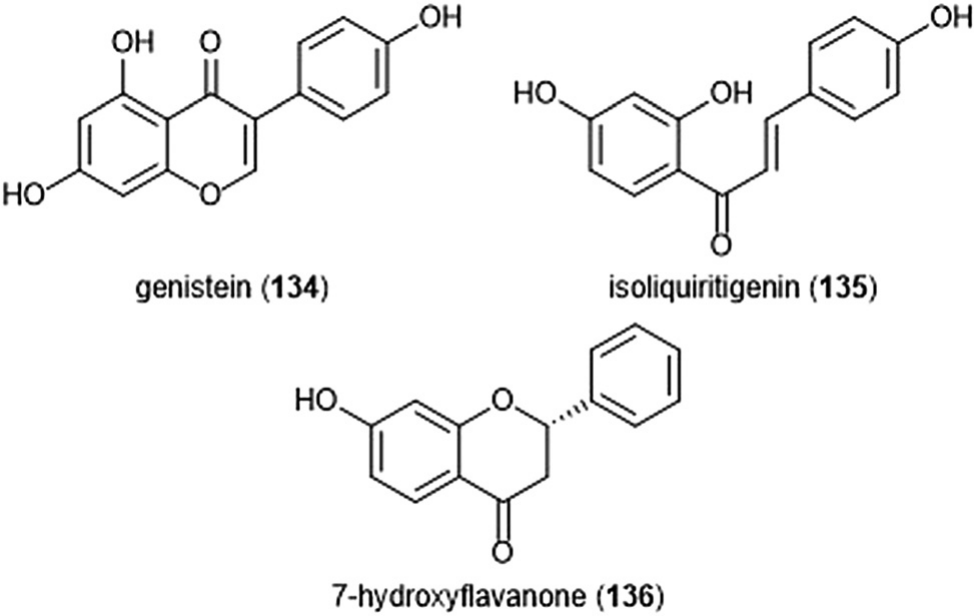
The flavonoids genistein, isoliquiritigenin and 7-hydroxyflavanone are shown.

**Chart 33:**
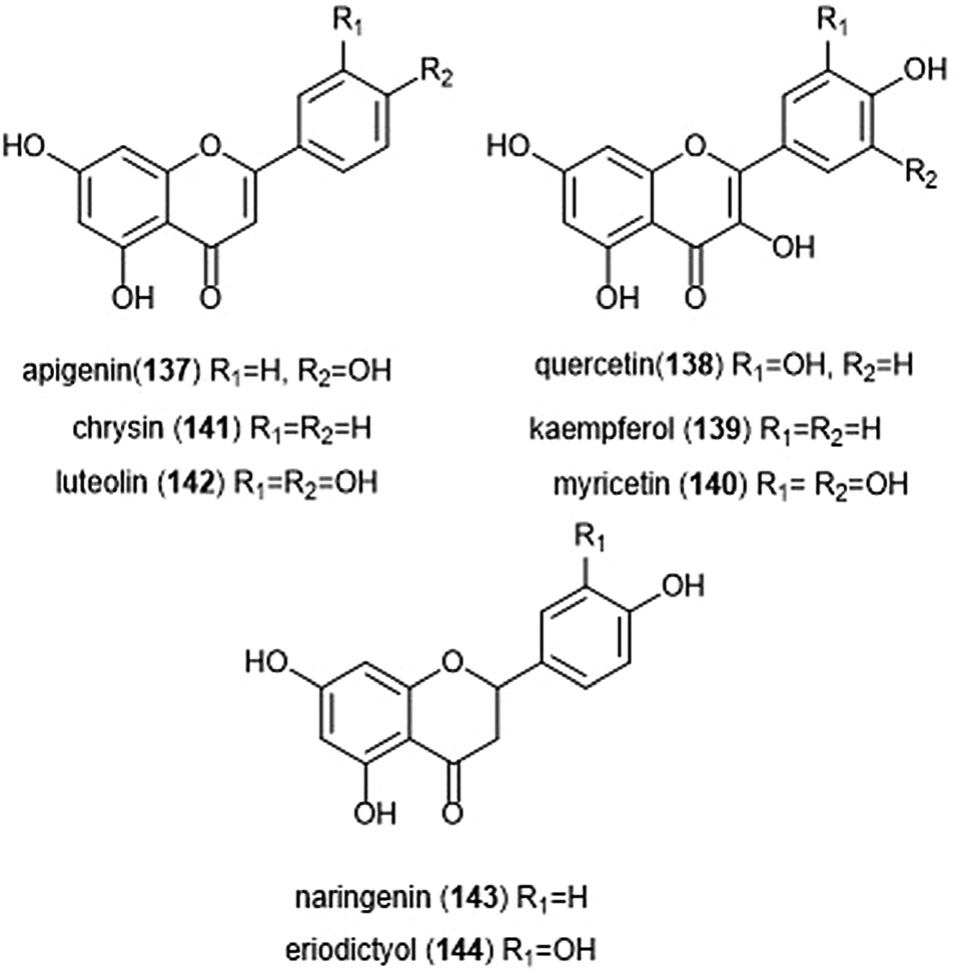
Various flavonoids with flavone, flavanol and flavanone substructures inhibit the 20S proteasome, and are depicted.

**Chart 34:**
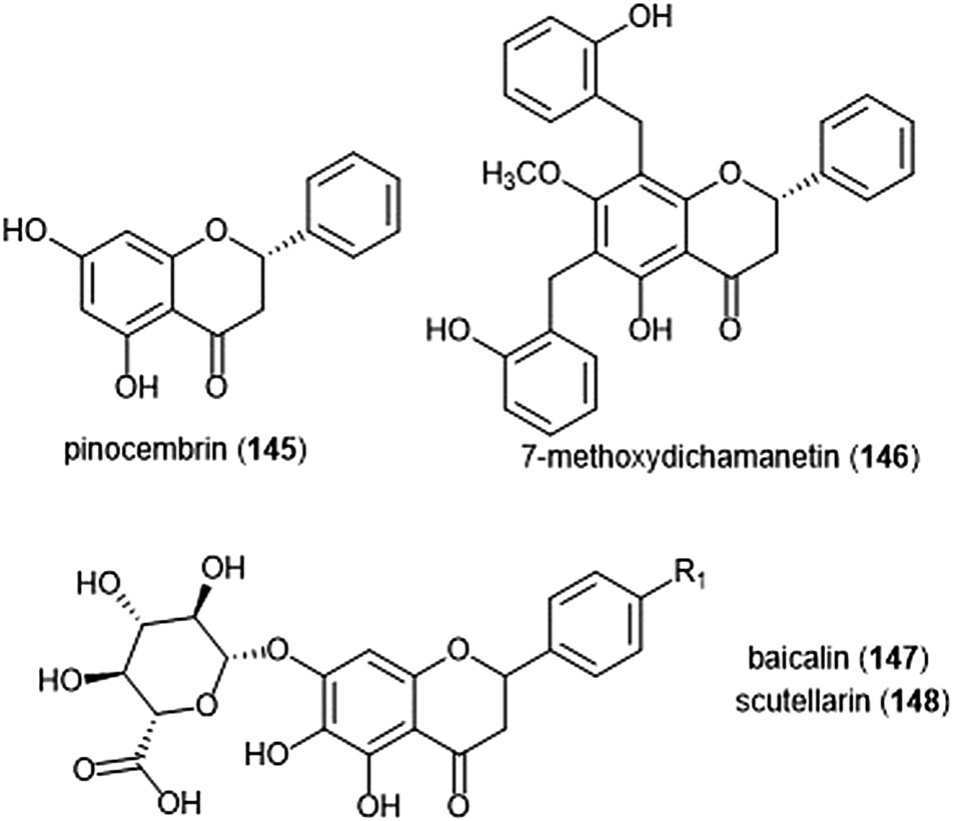
Several flavanone-containing proteasome inhibitors have been discovered and are shown.

**Chart 35:**
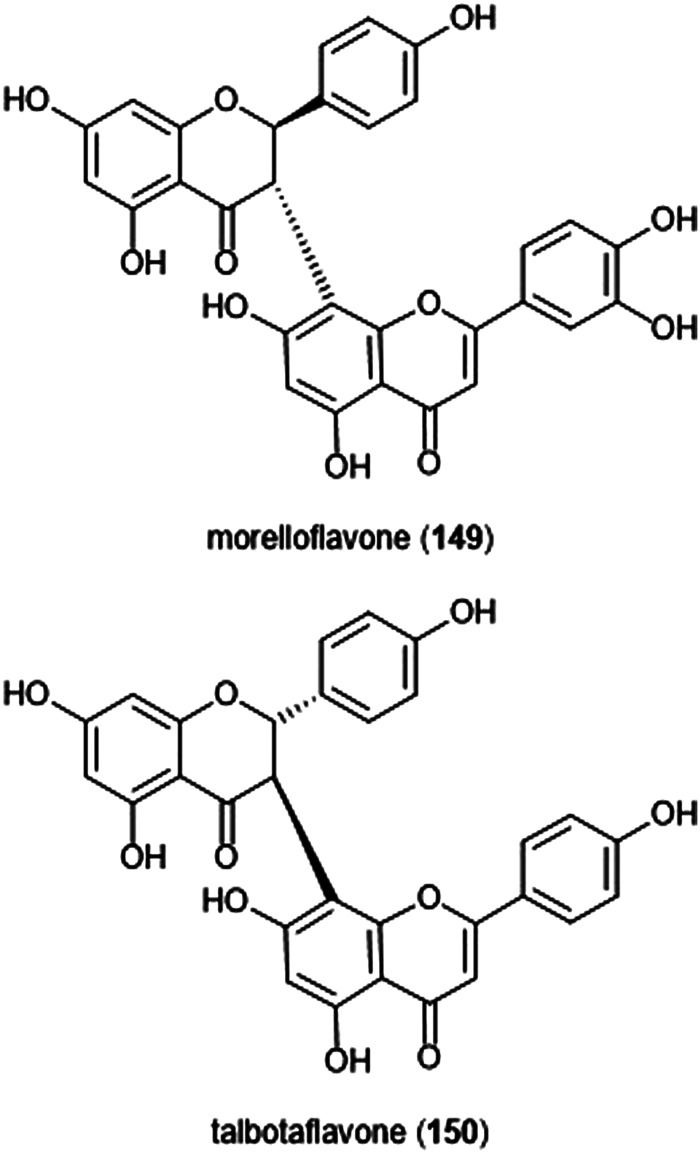
The biflavonoid-containing proteasome inhibitors morelloflavone and talbotaflavone are shown.

**Chart 36:**
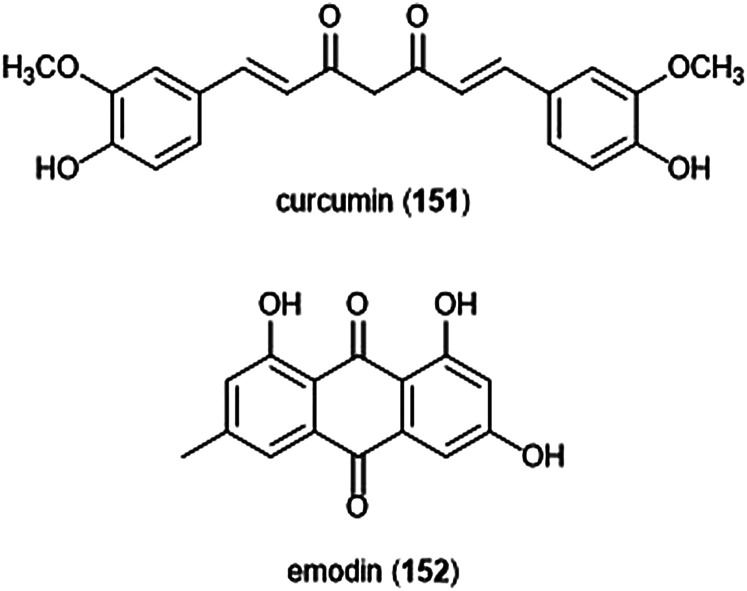
The flavonoids curcumin and emodin are shown.

In addition to the tea polyphenols, several other flavonoids have been identified for their ability to inhibit the proteasome (Charts 32–36). Specific features of the flavonoids contribute to the interactions between the compounds and target active sites. The soy isoflavone genistein (**134**) was first reported in 2003 by Kazi *et al.* for its inhibitory activity towards the ChT-L site of the 20S proteasome in a cell-free assay with an IC_50_ value of 26 μM.^247^ Docking studies suggested that unlike tea polyphenol (—)-EGCG, genistein does not covalently interact with the Thr1 residue of the active site. Genistein was also isolated in addition to proteasome inhibitors isoliquiritigenin (**135**) and 7-hydroxyflavanone (**136**) from the plant *Spatholobus suberectus*.^248^ The three compounds exhibited low micromolar inhibition towards the β5-site of the 20S proteasome, with IC_50_ values ranging from 4.88 (±1.55) to 9.26 (±1.2) μM. Apigenin (**137**), quercetin (**138**), kaempferol (**139**) and myricetin (**140**) were later evaluated by Chen *et al.* for their ability to inhibit the 20S proteasome.^249^ All four compounds inhibited the ChT-L site of the 20S proteasome in a cell-free assay, with apigenin displaying the most potent activity (IC_50_: 1.8 ± 0.03 μM). The compounds displayed similar inhibitory activity towards the 26S proteasome in intact Jurkat cells. Apigenin was still the most potent of the four compounds. *In silico* docking studies suggested that the carbonyl at C-4 is the site of nucleophilic attack by the Thr1O^γ^ residue of the active site. Additionally, the hydroxyl group at C-3 was believed to interfere with binding of the flavonoids to the active site. Further studies by the same group evaluated the structure–proteasome–inhibitory activity relationship of flavonoids chrysin (**141**), luteolin (**142**), naringenin (**143**), and eriodictyol (**144**) in comparison to apigenin.^250^ The 2,3 double-bond featured within the flavanones chrysin, apigenin and luteolin was deemed necessary for of inhibition towards the ChT-L site of the proteasome. A subsequent study by Wu and Fang reported that the inhibition of chrysin, apigenin and luteolin is selective for ChT-L and T-L catalytic activities of the proteasome within tumor cells.^250^

**Chart 37:**
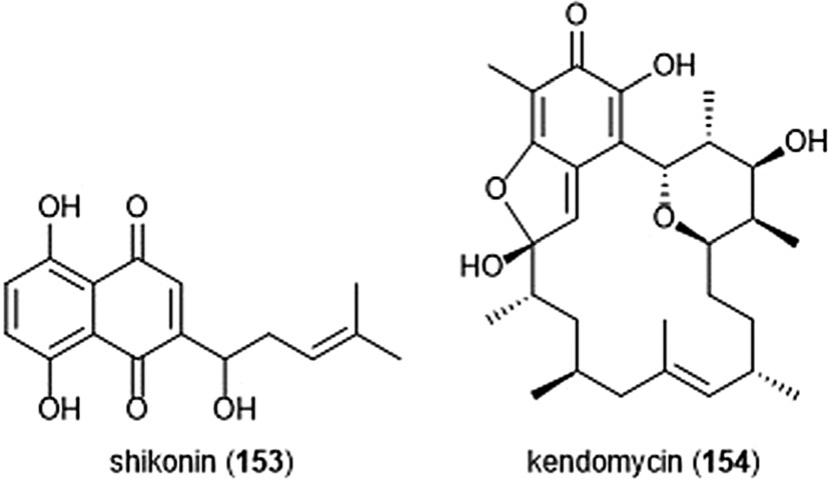
The polyketides shikonin and kendomycin have also been identified as proteasome inhibitors.

Pinocembrin (**145**) and 7-methoxydichamanetin (**146**) were recently isolated in a bioassay-guided fractionation of the *Piper sarmentosum* plant by Pan *et al.*^251^ These flavanones exhibited inhibitory activities toward the ChT-L site of the human 20S proteasome, with IC_50_ values of 2.87 ± 0.26 and 3.45 ± 0.18 μM, respectively. The flavonoid glycosides baicalin (**147**) and scutellarin (**148**) were isolated from the Chinese herbal medicines *Scutellaria baicalensis* and *Erigeron breviscapus* (Vant.) Hand-Mazz by Wu *et al.* These compounds exhibited the ability to preferentially inhibit ChT-L activity in A549 and HL60 cancer cells.^252^ The biflavonoids morelloflavone (**149**) and talbotaflavone (**150**) were isolated from the stem bark of *Garcinia lateriflora* by Ren *et al.*, and exhibited inhibition against the ChT-L site of the 20S proteasome, with IC_50_ values of 1.3 μM and 4.4 μM, respectively.^253^ The prenylated flavonoid sanggenon C was also reported as an inhibitor of the 20S proteasome.^254^ Huang *et al.* indicated that the natural product inhibits the ChT-L activity of the 20S proteasome in enzymatic studies and also in H22 cell lysate in a dose-dependent manner.

The promiscuous active agent curcumin (**151**) is a symmetric polyphenol which was also reported as a proteasome inhibitor through *in vitro* and *in vivo* studies.^255,256^ Curcumin inhibits the ChT-L activity of the 20S mammalian proteasome with an IC_50_ value of 1.85 μM, demonstrates the ability to inhibit the 26S proteasome in human colon cancer HCT-116 and SW480 cell lines, and also induces apoptosis. *In silico* docking studies suggests that the ketone moieties are susceptible to nucleophilic attack by the Thr1O^γ^ of the chymotrypsin-like site. A recent study by Zhang *et al.* introduced an α-aminoboronic acid electrophile to the scaffold to improve potency.^257^ These compounds displayed impressive growth inhibitory activity against HCT-116 cells.

Emodin (**152**) was recently identified as an inhibitor of the 26S proteasome in the HEK293A-luciferase-cODC cell line. Emodin inhibited luciferase-ODC degradation with an EC_50_ of 6.33 μM and also exhibited inhibitory activity against the ChT-L and C-L sites of the proteasome, with IC_50_ values of 1.22 and 0.24 μM, respectively.^258^*In silico* docking with the catalytic sites of the proteasome indicated that the carbonyls are susceptible to nucleophilic attack by the Thr1O^γ^ much like the other carbonyl-containing flavonoids.

### Polyketides

Polyketides represent another class of natural products which have been scrutinized for their biological activity (Chart 37). The napthoquinone shikonin (**153**) was isolated from the traditional Chinese medicine *Zi Cao* (*gromwell*) and reported as an inhibitor of ChT-L activity of 20S rabbit proteasome (IC_50_: 12.5 μM).^259^ The natural product also demonstrates inhibitory activity towards the 26S proteasome in cell studies (PC-3 and murine hepatoma H22). A later study by Wada *et al.* complemented these results, demonstrating the ability of shikonin to induce apoptosis in various multiple myeloma cells including bortezomib resistant cells KMS11/BTZ (SHK at 2.5–5 μM).^260^*In silico* docking studies suggest that the quinone carbonyls interact with the ChT-L site in such a way that they became highly susceptible to the catalytic site's nucleophilic Thr1 residue.

Recently, the cytotoxic macrocycle kendomycin (**154**) was identified as a weak inhibitor of the proteasome.^261^ Kendomycin exhibited the ability to weakly inhibit the activity of the ChT-L site of the proteasome (IC_50_: 67.9 μM). X-ray crystallographic analysis of the yCP: kendomycin complex indicates that it does not interact with the inner chamber of the core particle, but rather covalently attaches to β2-H141N^γ^ along the outside of the 20S proteasome. Kendomycin sits within the surface-exposed pocket formed by the interface of the β2–β7′ subunits.

### Macrolides

**Chart 38:**
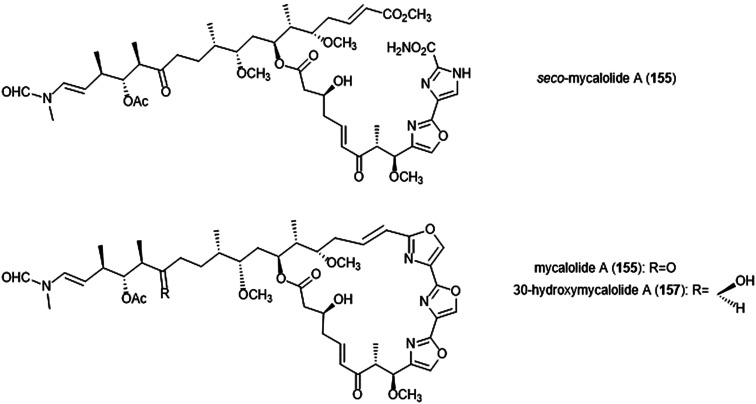
Mycalolide B and its natural analogs seco-mycalolide A and 30-hydroxymycalolide A have been identified as inhibitors of the 20S proteasome, and their structures are displayed.

*Seco*-mycalolide A (**155**) was isolated alongside known mycalolide A (**156**) and 30-hydroxymycalolide A (**157**) from a marine sponge of the genus *Mycale* (Chart 38). The mycalolides had previously been reported for their cytotoxicity towards B-16 melanoma cells. *In vitro* studies indicated that these compounds inhibit the ChT-L activity of the proteasome, with IC_50_ values of 11.5, 33.0 and 49.4 μM, respectively.^262^ These results suggested that the intact macrocycle is not necessary for inhibitory activity, which could allow for the design of simplified analogues in future studies.

**Chart 39:**
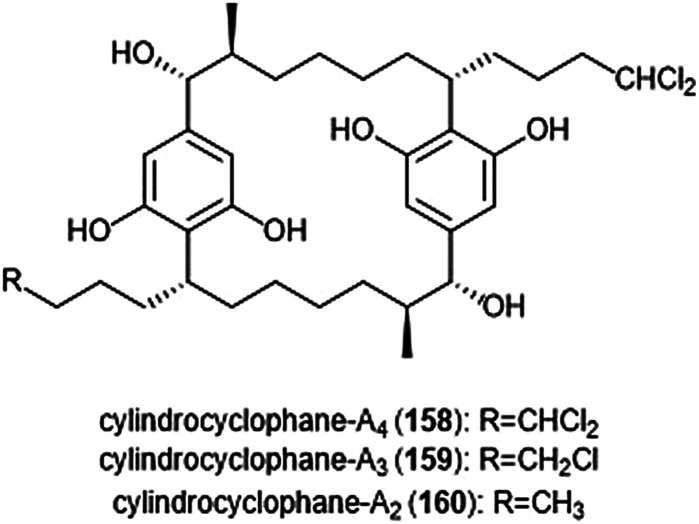
The cylindrocyclophanes A2 and A4 are depicted.

Several macrocyclic cylindrocyclophanes were also reported as proteasome inhibitors by Chlipala *et al.*^263^ The compounds were isolated from the extract of the terrestrial cyanobacteria *Nostoc* species (UIC 10022A) which was collected from a Chicago city parkway. The most potent cylindrocyclophanes A_4_–A_2_ (**158–160**) inhibit the ChT-L activity at low micromolar concentrations (IC_50_: 3.93 ± 0.18, 2.75 ± 0.31, and 2.55 ± 0.11 μM, respectively). Cylindrocyclophanes A_4_–A_2_ also display potent cytoxicity against HT-29 cells, with EC_50_ values of 2.0, 0.5 and 1.7 μM, respectively. Researchers suggested that in addition to a dichloromethyl moiety, the presence of hydroxyl group at C14 was also important for inhibitory activity. However, the inhibitory potency results did not correlate to cytotoxicity results, suggesting that the main reason for cytotoxicity was not due to proteasome inhibition (Chart 39).

**Chart 40:**
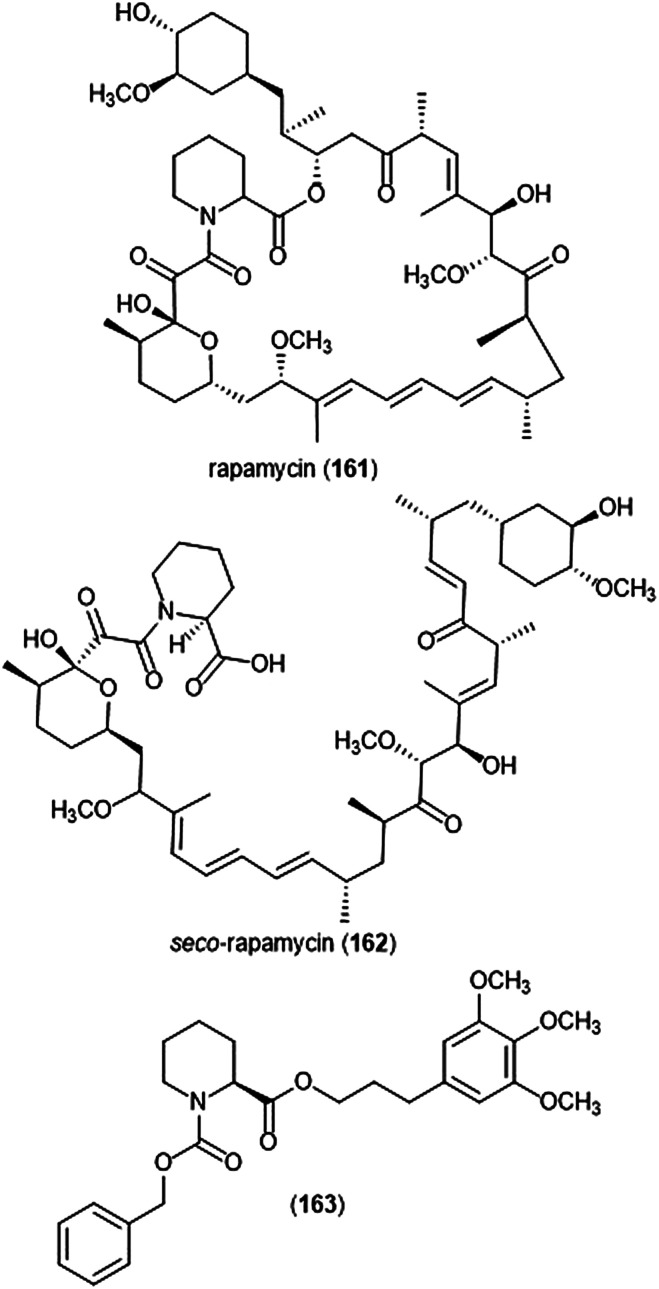
Rapamycin and its natural and synthetic analogs are shown.

The macrolide rapamycin (**161**), its analogues, and its acyclic analog *seco*-rapamycin (**162**) have also been identified as allosteric inhibitors of the 20S proteasome by Osmulski and Gaczynksa (Chart 40).^264^ Rather than bind competitively within the catalytic sites of the 20S proteasome, these compounds bind to specific grooves on the α-ring to confer inhibition. Rapamycin inhibits the ChT-L and T-L activities of the 20S proteasome in a reversible manner, with IC_50_ values of 1.9 and 0.4 μM, respectively. Identification of a minimal binding pharmacophore led researchers to the discovery of analog **163**, a pipecolic ester carbamate which inhibits the ChT-L site of the 20S proteasome (IC_50_ value of 2.0 μM).^265^

## Conclusions

Modulation of the 20S proteasome is a valuable strategy for the treatment of many diseases. Several natural product classes have been identified as proteasome inhibitors, making them intriguing starting points in the search of drug leads. Not only do these scaffolds provide opportunity for inhibition by allosteric and competitive modes, the complex structures have also facilitated extensive structure–activity relationship studies to better understand their mechanism of interaction with the 20S proteasome. Inherent challenges associated with the use of natural product-based inhibitors include product isolation from crude mixtures and subsequent synthesis of the complex substrates, as exemplified in the case of some natural product-based inhibitors. However, the use of X-ray crystallography and computational docking have been integral in the determination of their mechanisms of inhibition. These methods allow for the strategic synthesis of novel (often simplified) scaffolds, which retain the key components of their parent molecule. Modification of natural product scaffolds by researchers has led to more potent, physiologically relevant, and selective inhibitors as starting points for the treatment of disease. Thus far, the clinical impact of proteasome inhibitors has been significant with several agents currently clinically used to treat multiple myeloma and mantle cell lymphoma.

## Conflicts of interest

There are no conflicts to declare.

## Supplementary Material
